# Elamipretide alleviates pyroptosis in traumatically injured spinal cord by inhibiting cPLA2-induced lysosomal membrane permeabilization

**DOI:** 10.1186/s12974-023-02690-4

**Published:** 2023-01-07

**Authors:** Haojie Zhang, Yituo Chen, Feida Li, Chenyu Wu, Wanta Cai, Hantao Ye, Haohan Su, Mingjun He, Liangliang Yang, Xiangyang Wang, Kailiang Zhou, Wenfei Ni

**Affiliations:** 1 Department of Orthopaedics, The Second Affiliated Hospital and Yuying Children’s Hospital of Wenzhou Medical University, Wenzhou, 325000 Zhejiang China; 2 The Second School of Medicine, Wenzhou Medical University, Wenzhou, 325000 Zhejiang China; 3 Key Laboratory of Orthopaedics of Zhejiang Province, Wenzhou, 325000 Zhejiang China; 4 School of Pharmaceutical Sciences, Wenzhou Medical University, Wenzhou, 325035 Zhejiang China

## Abstract

Spinal cord injury (SCI) is a devastating injury that may result in permanent motor impairment. The active ingredients of medications are unable to reach the affected area due to the blood‒brain barrier. Elamipretide (SS-31) is a new and innovative aromatic cationic peptide. Because of its alternating aromatic and cationic groups, it freely crosses the blood‒brain barrier. It is also believed to decrease inflammation and protect against a variety of neurological illnesses. This study explored the therapeutic value of SS-31 in functional recovery after SCI and its possible underlying mechanism. A spinal cord contusion injury model as well as the Basso Mouse Scale, footprint assessment, and inclined plane test were employed to assess how well individuals could function following SCI. The area of glial scarring, the number of dendrites, and the number of synapses after SCI were confirmed by HE, Masson, MAP2, and Syn staining. Western blotting, immunofluorescence, and enzyme-linked immunosorbent assays were employed to examine the expression levels of pyroptosis-, autophagy-, lysosomal membrane permeabilization (LMP)- and MAPK signalling-related proteins. The outcomes showed that SS-31 inhibited pyroptosis, enhanced autophagy and attenuated LMP in SCI. Mechanistically, we applied AAV vectors to upregulate *Pla2g4A* in vivo and found that SS-31 enhanced autophagy and attenuated pyroptosis and LMP by inhibiting phosphorylation of cPLA2. Ultimately, we applied asiatic acid (a p38-MAPK agonist) to test whether SS-31 regulated cPLA2 partially through the MAPK-P38 signalling pathway. Our group is the first to suggest that SS-31 promotes functional recovery partially by inhibiting cPLA2-mediated autophagy impairment and preventing LMP and pyroptosis after SCI, which may have potential clinical application value.

**Supplementary Information:**

The online version contains supplementary material available at 10.1186/s12974-023-02690-4.

## Introduction

Spinal cord injury (SCI) is a serious and potentially fatal condition that may result in the loss of motor and sensory function [1]. Every year, between 250,000 and 500,000 individuals worldwide suffer from SCI [2]. Clinical therapy for SCI is extremely difficult; the only two medications for SCI that have been clinically shown to be useful are ganglioside and methylprednisolone [3]. Thus, it is important to understand what causes SCI and how to treat it effectively. SCI pathophysiology is divided into two parts: primary and secondary injuries. A lengthy period of secondary injury follows the primary short-term, direct mechanical injury to the tissues. Secondary damage manifests as neuroinflammation, ischaemia, oxidative stress, and cell death [4, 5]. Current evidence indicates that inhibition of neuronal death and promotion of cellular organelle integrity are vital for SCI [6, 7].

Research on programmed cell death has made some very encouraging recent developments, opening up new possibilities for SCI treatment. In recent years, it has been demonstrated that the programmed cell death process known as pyroptosis, which promotes inflammation, substantially influences neuroinflammation following SCI [8]. Mechanistically, there are two forms of pyroptosis: canonical (regulated by caspase-1) and noncanonical. Toll-like receptors and Nod-like receptors search for molecular patterns associated with danger or infections within the "priming stage" of classical pyroptosis, which is dependent on caspase-1. Molecular patterns associated with danger or pathogens are identified by Toll-like receptors and Nod-like receptors [9]. Once this occurs, inflammasome-related genes are more inclined to be activated, resulting in the production of pro-IL-1β and pro-IL-18. Later, the inflammasome forms, and caspases are activated [10]. Inflammasomes are formed by binding of pro-caspase-1 with adaptor protein apoptosis-associated speck-like proteins (often with the help of NLRP3). Then, pro-caspase-1 is cleaved to become caspase-1, which aids in the division of pro-IL-1β/18 as well as the fragmentation of GSDMD into two pieces [11]. The N-terminal fragment creates 10-15 nm holes within the cell membrane, which ultimately causes inflammatory factors to be released, cells to enlarge, and the membrane to break [12]. In summary, pyroptosis is the critical pathological mechanism after SCI and is regarded as an uncontrollable and unregulated process throughout the subacute period of SCI [13]. Therefore, inhibiting the occurrence and development of pyroptosis in neurons is vital for treating SCI.

Autophagy, a lysosome-dependent catabolic pathway, is also a form of cell death. Unlike pyroptosis, autophagy can degrade cytoplasmic proteins and organelles, which is important for maintaining cell homeostasis and preventing neurodegeneration [14]. The process of autophagy involves the formation of the phagophore, the development of autophagosomes, the fusion of autophagosomes with lysosomes to generate autophagic lysosomes, and the subsequent degradation of autolysosomes [15]. It is obvious that lysosomes are clearly required for autophagic flux. Lysosomal dysfunction, particularly an increase in lysosomal membrane permeability, has been associated with ageing and a variety of neurodegenerative diseases [16, 17, 18]. Lysosomal membrane permeabilization (LMP) leads to spillage of lysosomal contents into the cytoplasm, which hinders autophagic flux and destroys neuronal cells [6, 19, 20]. Maintaining the normal permeability of the lysosomal membrane is crucial for maintaining lysosomal function as well as shielding cellular components from lysosomal luminal enzyme exposure. Thus, prevention of LMP could benefit the restoration of autophagic flux and ultimately ameliorate SCI.

SS-31 (D-Arg-2', 6'-dimethyl-tyrosine-Lys-Phe-NH2), also known as elamipretide, is a short tetrapeptide that travels to the mitochondria, synthesized by Hazel H. Szeto and Peter W. Schiller [21]. It has a dimethyl-tyrosine residue that allows it to eliminate oxyradicals and prevent the oxidation of linoleic acid and low-density lipoprotein [21]. The alternating aromatic and cationic group structure of SS-31 allows it to readily cross the blood‒brain barrier [21] and subsequently enter the central nervous system (CNS), thus exhibiting a powerful protective effect against CNS diseases [22, 23, 24, 25]. For instance, after protracted neuroinflammation in aged rats, SS-31 improved autophagy to enhance functional connectivity within the hippocampus and other associated locations [23, 26]. Similar research has demonstrated that SS-31 largely reduces postoperative neurocognitive impairments in old mice by reducing pyroptosis [27]. Additionally, ischaemia is a key pathophysiological mechanism following SCI [28], and prior research in preclinical models has demonstrated that SS-31 protects mitochondrial integrity in ischaemia‒reperfusion injury connected to acute kidney injury [22] or hind limb paralysis [29]. These findings suggested that SS-31 use could have a positive impact on SCI. The function of SS-31 in SCI, nevertheless, has never been explored. Interestingly, increasing evidence suggests that cytosolic phospholipase A2 (cPLA2) activates the mitogen-activated protein kinase (MAPK) signalling pathway and plays a significant role in the development of SCI [19, 30], while prior research has demonstrated that SS-31 exerts its effects by inhibiting the p38-MAPK signalling pathway [31, 32]. Here, we employed a contusion SCI model in mice to verify the therapeutic effect of SS-31. Specifically, we sought to ascertain (1) whether SS-31 has a neuroprotective and motor recovery effect in SCI; (2) whether LMP, pyroptosis and autophagy are involved in the mechanism of SS-31 treatment in SCI; (3) whether cPLA2 is a mediator of the effect of SS-31 in SCI; and (4) whether SS-31 exerts its effects through the MAPK signalling pathway (or one of the MAPK pathways).

## Materials and procedures

### Animals and ethics statement

Female animals are used for most SCI experimental studies, as they have shorter urethras than male mice; thus, artificial voiding for urine retention after SCI and overall care is easier with females [33, 34]. Female adult C57BL/6 mice with an average weight of 20–30 g were obtained from Wen-Zhou Medical University’s Experimental Animal Center (licence no. SCXK [ZJ] 2020-0001), Zhejiang Province, China. All of the mice were kept in typical settings (21–25 °C, 12-h light/dark cycle, 50–60% humidity) with unlimited access to food and water. The experimental procedure related to animals followed the China National Institutes of Health’s Guide for the Care and Use of Laboratory Animals and was authorized by Wenzhou Medical University’s Animal Care and Use Committee (wydw 2017‒0096).

### Antibodies and reagents

Topscience in Shanghai, China, manufactured the SS-31 (TP1096) used in this study. Solarbio Science & Technology in Beijing, China, supplied the pentobarbital sodium, the Masson staining instrument, and the haematoxylin and eosin (HE) staining tools. Sigma‒Aldrich in St. Louis, Missouri, US, supplied chloroquine (CQ, Catalogue Number: C6628), asiatic acid (AA, Catalogue Number: A2612), a protease inhibitor cocktail (Catalogue Number: P8340), phosphatase inhibitor cocktail III (Catalogue Number: P0044) and the primary antibody against PLA2G4A (Catalogue Number: SAB4503812). Applied Biological Materials in Jiangsu, China, developed the Pla2g4a AAV Virus (Mouse) (CMV) (serotype# 1, with no fluorescent reporter gene) and AAV Blank Control Virus (CMV) (serotype# 1, with no fluorescent reporter gene). Thermo Fisher Scientific in Waltham, US, supplied the Lysosome Enrichment Kit for Tissue & Cultured Cells (Catalogue Number: 89839). Cell Signaling Technology in Beverly, Massachusetts, US, provided the primary antibodies against Beclin-1 (Catalogue Number: 3738), LC3B (Catalogue Number: 3868), NLRP3 (Catalogue Number: 15101), ATP6V1B2 (Catalogue Number: 14617), p38 (Catalogue Number: 8690t), and p-p38 (Catalogue Number: 4511t). The Proteintech Group in Chicago, Illinois, US, produced the VPS34 (Catalogue Number: 12452-1), CTSD (Catalogue Number: 21327-1), CASP1 (Catalogue Number: 22915-1), PLA2G4A (Catalogue Number: 18088-1-AP), and GAPDH (Catalogue Number: 10494-1) antibodies. Abcam in Cambridge, UK, provided the goat anti-mouse IgG H&L (Alexa Fluor® 594) (Catalogue Number: ab150116), goat anti-mouse IgG H&L (Alexa Fluor® 488) (Catalogue Number: ab150113), goat anti-rabbit IgG H&L (Alexa Fluor® 488) (Catalogue Number: ab150077), goat anti-rabbit IgG H&L (Alexa Fluor® 594) (Catalogue Number: ab150080), ASC (Catalogue Number: ab180799), synaptophysin (Syn) (Catalogue Number: 32127), microtubule-associated protein-2 (MAP2) (Catalogue Number: ab5392), mouse monoclonal to NeuN (Catalogue Number: ab104224), rabbit monoclonal to NeuN (Catalogue Number: ab177487), p62/SQSTM1 (Catalogue Number: ab240635), CTSB (Catalogue Number: ab214428), Myelin Basic Protein (MBP) (Catalogue Number: ab218011) and LAMP1 (Catalogue Number: ab24170) antibodies. R&D Systems in Minnesota, US, provided the CTSL (Catalogue Number: AF1515) antibody. Abclonal Technology in Cambridge, MA, US, provided IL-1β (Catalogue Number: A1112) and IL-18 (Catalogue Number: A1115) antibodies. Affinity Biosciences in Ohio, US, provided the NLRP1 (Catalogue Number: DF13187), GSDMD-N (Catalogue Number: DF12275), extracellular signal-regulated kinase 1/2 (ERK1)/2 (Catalogue Number: AF0155), p-ERK1/2 (Catalogue Number: AF1015), c-Jun N-terminal kinase (JNK) (Catalogue Number: AF6318), and p-JNK (Catalogue Number: AF3318) antibodies. Santa Cruz Biotechnology in Dallas, Texas, US, provided the horseradish peroxidase (HRP)-conjugated IgG secondary antibody and the cPLA2 (Catalogue Number: sc-376618), GFAP (Catalogue Number: sc-166458), and Iba-1 (Catalogue Number: sc-32725) antibodies. Boyun Biotechnology in Nanjing, China, supplied the fluorescein isothiocyanate (FITC)-conjugated IgG secondary antibody. The 4′,6-diamidino-2-phenylindole (DAPI) solution was provided by Beyotime Biotechnology in Jiangsu, China. EIAab in Wuhan, China, supplied the PLA2G4A ELISA kit (Catalogue Number: E1624m) and the CTSD ELISA kit (Catalogue Number: E1280m). MSKBIO Company in Wuhan, China, supplied the alpha-N-acetylglucosaminidase (NAGLU) ELISA kit (MSKBIO, KT-22858).

### Animal model of SCI

Prior to the operation, each animal was given 50 mg/kg of 1% (w/v) pentobarbital sodium intraperitoneally for anaesthesia. The T9–T10 vertebrae were then subjected to a conventional laminectomy to reveal a circle of the dura. The mice with exposed spinal cords were then subjected to a spinal cord weight drop. Briefly, a 5 g weight was dropped from a height of 30 mm to strike the exposed spinal cord and induce mild SCI, as advised by the device manufacturer (W.M. KECK+, Model+III, US) [35]. Thereafter, 4-0 silk and a needle were used to stitch the muscle and skin in layers. Three times per day after SCI, the mice were manually urinated until the bladder reflex returned. The mice in the Sham group received anaesthesia and a laminectomy without suffering any spinal cord damage.

### Adeno-associated virus (AAV) vector packaging

AAV-Pla2g4a (mouse *Pla2g4e*) and AAV-Blank (mouse *Blank*) were constructed and packaged by Applied Biological Materials Company Co., Ltd. (Jiangsu, China). The detailed protocols were conducted according to previously published instructions [36, 37]. Quantitative PCR (qPCR) demonstrated that AAV-Pla2g4a had 5.63×10^12^ genomic copies per mL and that AAV-Blank had 8.74×10^12^ genomic copies per mL.

### AAV vector injection

AAVs may need several days to take effect in vivo, and according to previous studies [7, 38], 2 weeks before SCI is considered an appropriate time point to carry out AAV injection. Therefore, 2 weeks before surgery, the Sham+AAV-Pla2g4a, SCI+AAV-Pla2g4a and SCI+AAV-Pla2g4a+SS-31 groups were injected with 2 µl of effective viral vectors in PBS at the T9–T10 level to a depth of 1.5 mm from the dorsal surface of the spinal cord with microsyringes. The Sham+AAV-Blank, SCI+AAV-Blank and SCI+AAV-Blank+SS-31 groups were injected with an equal volume of the blank viral vectors. After injection, the needle was left in place for 1 min before removal to prevent leakage of the virus/vehicle. Following injection, the muscle and skin over the exposed spinal cord were sutured. Later, all mice were placed on a warm blanket for postoperative recovery. No mice exhibited hind limb paralysis or paresis after injection.

### Treatment and groups

The mice were separated randomly into 13 groups: the Sham (*n* = 30), Sham+SS-31 (*n*=10), Sham+CQ (*n*=10), Sham+AAV-Blank (*n *= 10), Sham+AAV-Pla2g4a (*n*=10), SCI (*n* = 45), SCI+SS-31 (*n* = 45), SCI+SS-31+CQ (*n* = 20), SCI+SS-31+AA (*n*=5), SCI+AAV-Blank (*n* = 20), SCI+AAV-Pla2g4a (*n* = 20), SCI+AAV-Blank+SS-31 (*n* = 20), and SCI+AAV-Pla2g4a+SS-31 (*n* = 20) groups. The Sham+SS-31, SCI+SS-31, SCI+SS-31+AA, SCI+SS-31+CQ, SCI+AAV-Blank+SS-31 and SCI+AAV-Pla2g4a+SS-31 groups were treated with SS-31 (5 mg/kg) via daily intraperitoneal injection for 30 min prior to surgery and for three consecutive days following SCI. The dose and time of SS-31 administration were adopted according to a previous review regarding SS-31 [23]. An equal volume of vehicle was administered to the Sham, SCI, SCI+AAV-Blank and SCI+AAV-Pla2g4a groups. Daily intraperitoneal injection of CQ (60 mg/kg) or asiatic acid (AA, 550 μg/kg) was performed 30 min prior to SS-31 administration for 3 days. The dose and time of CQ and AA administration were chosen according to previous studies on CNS trauma [39, 40]. The mice were killed via an overdose of pentobarbital sodium, and histological specimens were obtained for relevant assays at 3 or 28 days post-injury (dpi).

### Evaluation of functional behaviour

On days 0, 1, 3, 7, 14, 21, and 28 following SCI, Basso Mouse Scale (BMS) score and the inclined plane test were performed. The BMS ranged from 0 to 9, with 0 indicating total paralysis and 9 indicating full motor function [41]. The inclined plane test was employed to determine the maximum angle at which mice could remain on the testing apparatus in two locations for at least 5 s without falling. Both of the above experiments were conducted in an open field. At 28 days after surgery, footprint analysis was conducted. Mouse forelimbs and hind limbs were stained separately with blue and red dye [42]. The footprints were used to measure the toe dragging and stride length. Toe dragging was calculated as the ratio of the whole length of the back leg drag to the whole walking length. The stride length was calculated as the distance between the adjacent hind limbs. Two impartial testers who were unaware of the experimental setup carried out the above experiment and measured the outcomes.

### Preparation of tissue slides for HE and Masson staining

On the 28th postoperative day, the mice were placed under deep anaesthesia and euthanized by transcardial perfusion with ice-cold 100 mM phosphate-buffered saline (PBS, pH 7.4) followed by 4% (w/v) paraformaldehyde (PFA) in PBS. The whole section (10 mm in length, with the epicentre in the middle) was then postfixed in 4% (w/v) PFA for 24 h. Every sample was embedded in paraffin and then prepared for longitudinal paraffin sectioning. For HE staining, 4-μm slices were cut via a microtome and mounted on poly-L-lysine-coated slides according to a previous study [43]. For Masson staining, we mordanted longitudinal sections with 10% potassium dichromate and 10% trichloroacetic acid, and we stained the nuclei with haematoxylin. The sections were differentiated with ethanol and hydrochloric acid, made blue again with mild ammonia and stained with Masson solution. This staining approach has been described previously [43]. Finally, images were acquired with an Olympus light microscope (Tokyo, Japan).

### Western blotting (WB)

On day 3, mice with SCI were euthanized, and 1 cm pieces of their spinal cords were cut out and mixed in an ice-cold RIPA lysis solution containing protease inhibitor cocktail and phosphatase inhibitor cocktail III. We utilized protein extraction reagents to extract all the proteins from the spinal cord samples. BCA assays were employed to measure the protein levels. Equal quantities of protein (60 μg) were isolated and transferred to PVDF films (Millipore) using 12% (w/v) gel electrophoresis. The films were blocked in 5% (w/v) skim milk for 2 h and probed with primary antibodies against the following proteins overnight at 4 °C: ASC (1:1000), NLRP1 (1: 1000), NLRP3 (1: 1000), IL-18 (1: 1000), IL-1β (1: 1000), GSDMD-N (1: 1000), Caspase-1 (1:1000), Beclin-1 (1:1000), SQSTM1/p62 (1:1000) LC3 (1:1000), VPS34 (1:1000), ATP6V1B2 (1:1000), CTSL (1:1000), CTSB (1:1000), CTSD (1:1000), LAMP1 (1:1000), GAPDH (1:1000), cPLA2 (1:1000), p-cPAL2 (1:1000), ERK1/2 (1:1000), p-ERK1/2 (1:1000), p38 (1:1000), p-p38 (1:1000), JNK (1:1000), and p-JNK (1:1000). The membranes were then incubated with HRP-conjugated IgG secondary antibodies for 2 h at room temperature. The band signals were observed and examined via a Bio-Rad ChemiDoc™ XRS+ Imaging System using an enhanced chemiluminescence (ECL) immune-detection instrument.

### Immunofluorescence (IF)

Slides of transverse spinal cord sections were prepared for IF via approaches that have been previously reported [44]. Following dewaxing and rehydration, the samples were fixed and subjected to high-pressure antigen retrieval. The slices were then blocked for 30 min at 37 °C with 5% bovine serum albumin in PBS and stained with primary antibodies against the following proteins overnight at 4 °C: MAP2 (1:200), Syn (1:200), NeuN (1:1000), Caspase-1 (1:200), NLRP3 (1:200), SQSTM1/p62 (1:200), LC3 (1:200), p-cPLA2 (1:200), CTSL (1:200), MBP (1:200), GFAP (1:200), and Iba-1 (1:200). The next day, the slices were reincubated at 37 °C for 1 h with secondary antibodies prior to being stained with DAPI stain solution. All images of transverse sections were captured 3 mm rostral to the lesion site, and the imaging results were assessed using a fluorescence microscope (Olympus, Japan) in 5 randomly acquired areas in the anterior horn from each specimen. The integrated density of MAP2, and Caspase-1, NLRP3, p62, p-cPLA2 in each neuron was calculated using ImageJ software. IF images of LC3 in each neuron were quantified to count specific LC3 II puncta with diameters larger than 0.2 but smaller than 10 μm, a size range consistent with autophagosomes [45]. The numbers of Syn-positive synapses on motor neurons were calculated manually in a double-blind manner. p-cPLA2-, NLRP3- and Caspase-1-positive cells (including microglia, astrocytes and oligodendrocytes) were also determined manually in a double-blind manner, and the number of positive cells was normalized to the total number of cells imaged.

### Quantitative PCR (qPCR)

Total RNA was extracted from the spinal cord via TRIzol reagent according to the supplier's instructions. Quantitative analyses were completed via a two-step reactive procedure: reverse transcription (RT) and PCR. Every RT reaction comprised 0.5 μg of RNA, 2 μl of 5× TransScript All-in-one SuperMix for qPCR and 0.5 μl of gDNA remover in an overall volume of 10 μl. The reaction was conducted via a GeneAmp® PCR System 9700 (Applied Biological Systems, America) at 42 °C for 15 min followed by 85 °C for 5 s. The 10 μl RT reaction mixture was then desaturated × 10 in nuclease-free water and held at − 20 °C. Real-time PCR was conducted via a LightCycler® 480 II Real-time PCR System (Roche, Switzerland) with 10 μl of a PCR mixture including 1 μl of cDNA, 5 μl of 2× PerfectStart^TM^ Green qPCR SuperMix, 0.2 μl of forward primer, 0.2 μl of reverse primer and 3.6 μl of nuclease-free water. The reaction process was cultivated in a well optic plate (Roche) at 94 °C for 0.5 min followed by 45 cycles at 94 °C for 5 s and 60 °C for 30 s. All specimens were analysed three times. After the PCR cycles, melting curve analyses were performed to verify the specific generation of the anticipated PCR products. The following primer sequences were developed in the laboratory and synthesized by TsingKe Biological Technology based on the mRNA sequences acquired from the NCBI database: *Sqstm1, 5ʹ- GATAGCCTTGGAGTCGGT-3ʹ (forward)* and *5ʹ-AAATGTGTCCAGTCATCGTC-3ʹ (reverse); Pla2g4a, 5ʹ-CAGCACATTATAGTGGAACACCA-3ʹ (forward)* and *5ʹ-AGTGTCCAGCATATCGCCAAA-3ʹ (reverse);* and *β-actin, 5ʹ-GGCTCCTAGCACCATGAAGA-3ʹ (forward)* and *5ʹ- AGCTCAGTAACAGTCCGCC -3ʹ (reverse).* Finally, the mRNA expression levels were normalized to those of *β-actin* and computed via the 2-ΔΔCt approach.

### Subcellular fractionation and preparation of lysosome-rich fractions

Spinal cord tissue fragments measuring 5 mm in length were gathered and homogenized on ice using a Dounce tissue grinder. Following the manufacturer's instructions (Lysosome Enrichment Kit for Tissue and Cultured Cells; Thermo Fisher Scientific, 89,839), differential centrifugation was used to obtain lysosomal fractions from tissue homogenates. The resulting supernatant fractions were saved as cytosolic fractions.

### ELISA

The activity of cPLA2 (EIAab, E1624 m), CTSD (EIAab, E1280 m), and NAGLU (MSKBIO, KT-22858) were determined with ELISA kits following the manufacturers’ instructions. The optical density of the samples was measured using a microplate reader at 550 nm with a correction wavelength of 450 nm for quantification of cPLA2, CTSD, and NAGLU.

### Statistics

Statistical analyses were performed with SPSS version 23.0. All data are presented as the means ± standard errors of the means (SEMs). All data presented in the study were normalized to control for unwanted sources of variation. Independent-sample *t* tests were employed to determine remarkably significant differences between two groups. One-way ANOVA based on LSD post hoc analysis or Dunnett’s T3 approach (equal variances not assumed) was conducted to assess significant differences between two groups among three groups. Two-way ANOVA followed by Tukey's multiple comparisons test were used to analyse differences among four or more groups when the data were normally distributed, and nonparametric Mann–Whitney *U* tests were used for groups if the data were not normally distributed. The BMS score and inclined plane test results at multiple time points were compared with repeated-measures ANOVA followed by an LSD test for between-group comparisons. We considered *P* < 0.05 or *P* < 0.01 to indicate statistical significance; ns = not significant. The symbols * and ** indicate *P* < 0.05 and *P* < 0.01, respectively.

## Results

### SS-31 promotes functional recovery after SCI

**Fig. 1:**
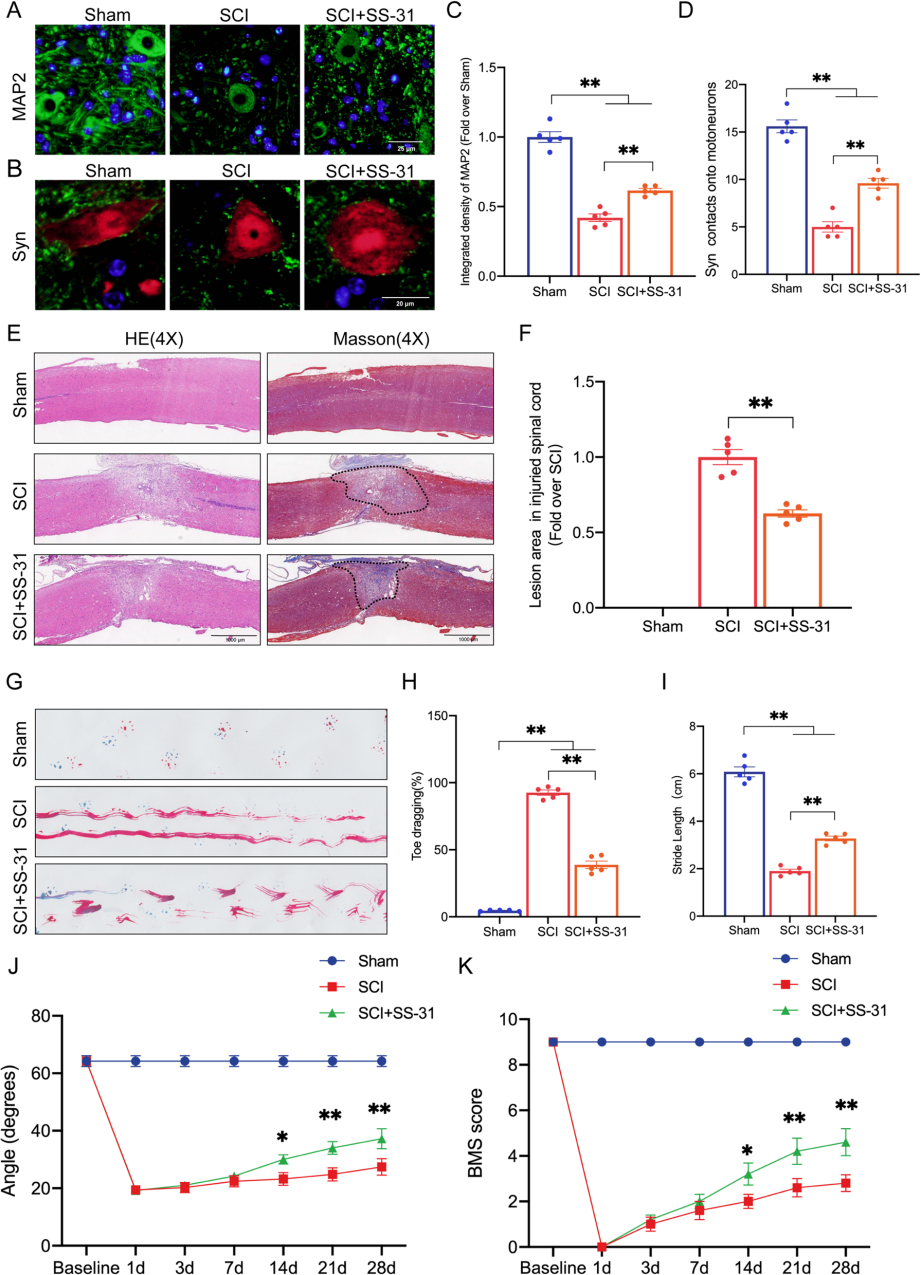
SS-31 facilitates functional recovery following SCI. **A** Photographs of spinal cord sections in the respective groups stained with antibody MAP2 (green) (scale bar = 25 μm). **B** Photographs of spinal cord sections in the respective groups stained with Syn (green)/NeuN (red) (scale bar = 20 μm). **C** MAP2 optical density within a spinal cord subjected to injury on day 28. **D** Relevant quantitative results for motor neuron-contacting synapse amounts on day 28 after SCI. **E** Longitudinal spinal cord sections from the groups at 28 dpi were examined via HE dyeing and Masson dyeing (scale bar = 1000 μm). **F** Quantitative investigations of Masson-positive lesions within the spinal cords of the respective groups. **G** Photographs of mouse footprints on day 28 following SCI. Blue: fore paw print; Red: hind paw print. **H**, **I** Toe dragging (%) and stride length (cm) analyses of mice at 28 dpi. **J**, **K** Inclined plane test and BMS results for the indicated groups at days 0, 1, 3, 7, 14, 21, and 28. The data are shown as the mean ± SEM. *n* = 5. *P < 0.05, **P < 0.01. ns indicates no significance

To evaluate the neuroprotective effect of SS-31 in mice with spinal cord contusions, we performed tissue staining and motor function evaluation. Synapsin (Syn) shows synaptic connectivity changes, the number of which can reflect motor cortical or spinal plasticity after traumatic injury [46]. A decrease in MAP2 in motoneurons distal to the lesion site indicates cytoskeletal abnormalities [47]. At 28 dpi, IF showed decreased MAP2 expression and fewer Syn-positive synapses on neurons in the SCI group than on neurons in the Sham group. The SCI mice treated with SS-31 had higher levels of neuronal MAP2 expression and more Syn-positive synapses than the SCI mice without any treatment (Fig. 1A–D). Additionally, the injured spinal cord lesion area was evaluated using HE and Masson staining, which revealed a glial scar area that was significantly expanded in the SCI group compared to the Sham group. However, SS-31 reduced the glial scar area in comparison to that in the SCI group (Fig. 1E, F). To further investigate the contribution of SS-31 to locomotive functional recovery, we performed footprint analysis, the inclined plane test and BMS score. Footprint analysis at 28 dpi showed significant gait recovery based on hind limb function in the SS-31+SCI group; in contrast, the mice in the SCI group remained unable to raise their hind limbs (Fig. 1G). The quantitative data measured by footprint analysis, including toe dragging and stride length, showed the same trends (Fig. 1H, I). Next, compared with the SCI group, the group of SS-31-treated SCI mice showed significantly higher BMS score and inclined plane angles at 14 dpi, 21 dpi and 28 dpi (Fig. 1J, K). In addition, we performed further experiments to determine the effects of SS-31 treatment in sham mice. As shown in Additional file 1: Fig. S1A–I, the MAP2 density, Syn numbers, toe dragging, stride length and BMS score exhibited no difference between non-SCI mice treated with and without SS-31. These results indicated that SS-31 treatment did not affect histological morphology and motor function recovery in non-SCI mice but effectively promoted functional recovery in SCI mice.

### SS-31 attenuates pyroptosis after SCI

**Fig. 2:**
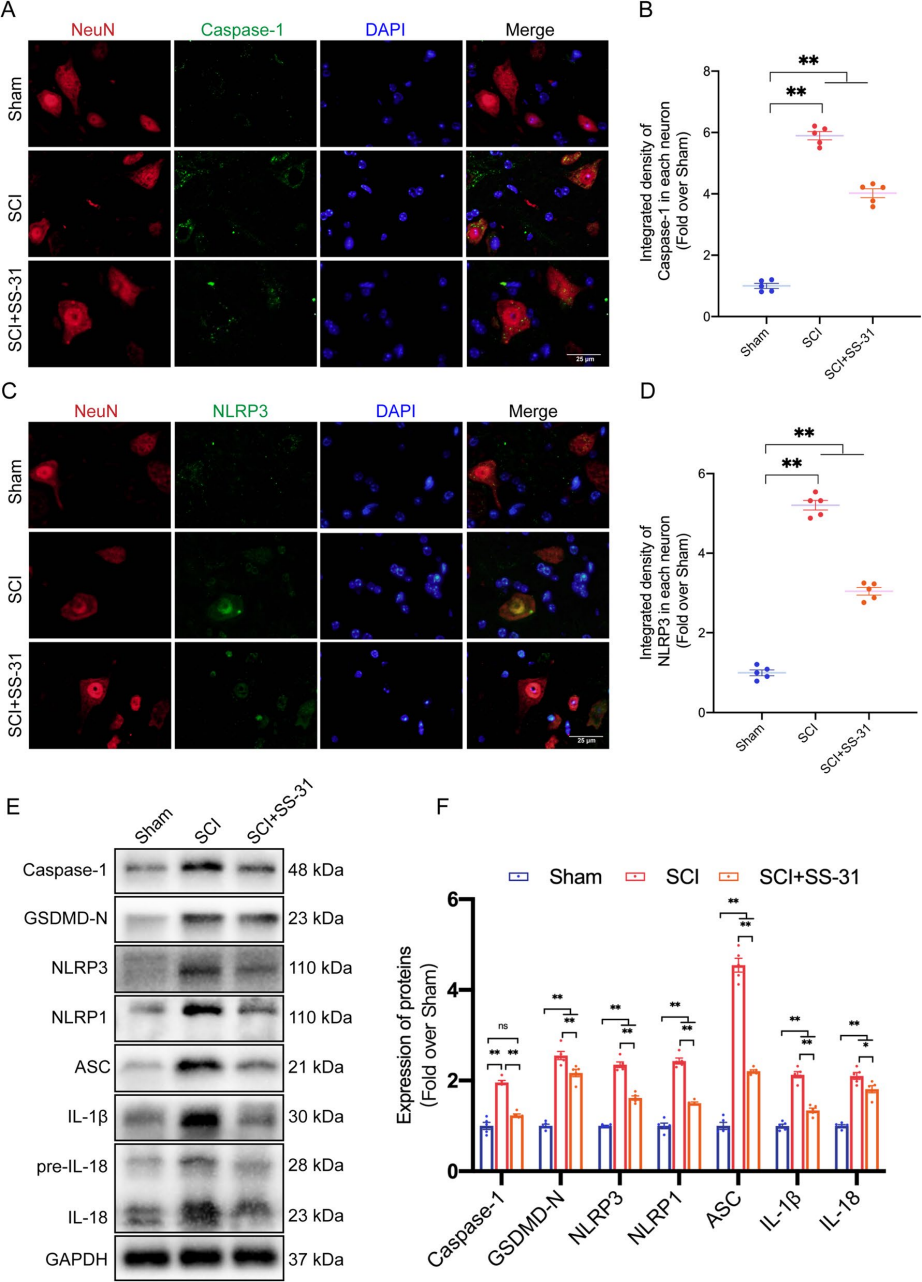
SS-31 attenuates pyroptosis after SCI. **A** Double-IF staining of Caspase-1 and NeuN in the spinal cords of the Sham, SCI, and SCI + SS-31 groups (scale bar = 25 μm). **B** Quantified Caspase-1 IF data are presented on the right. **C** Double-IF staining of NLRP3 and NeuN in the spinal cords of the indicated groups (scale bar = 25 μm). **D** Quantified NLRP3 IF data are presented on the right. **E** WB for Caspase-1, GSDMD-N, NLRP3, NLRP1, ASC, IL-1β, IL-18 and their expression levels in the Sham, SCI, and SCI + SS-31 groups. GAPDH was utilized as a loading control. The gels were run under the same experimental conditions, and the cropped blots are shown here. **F** The optical density values of Caspase-1, GSDMD-N, NLRP3, NLRP1, ASC, IL-1β and IL-18 were quantified and analysed in each group. The data are shown as the mean ± SEM. *n* = 5. *P < 0.05, **P < 0.01. ns indicates no significance

After traumatic tissue injury, inflammation is unavoidable. However, inflammation is a double-edged sword: rapid resolution of inflammation is necessary to ensure effective host defence and appropriate cell repair following an injury, but excessive inflammation induces further cell death, aggravating the injury [48]. A proinflammatory type of regulated cell death known as pyroptosis is mediated by cysteine-dependent aspartate-specific inflammatory proteases [48]. Pyroptosis has recently attracted considerable attention in relation to its specific function in inflammation. To determine whether SS-31 inhibits pyroptosis, we examined Caspase-1, GSDMD-N, NLRP3, NLRP1, ASC, IL-1β, and IL-18, which are essential for pyroptosis. IF staining of the SCI group revealed that Caspase-1 and NLRP3 fluorescence signals were strong in neurons. The SCI+SS-31 group, on the other hand, showed modest fluorescence signals (Fig. 2A–D). Notably, extensive evidence indicates that microglia-mediated pyroptosis is also a significant cause of neuroinflammation after SCI [13, 49]. Through IF staining, the numbers of Caspase-1- and NLRP3-positive microglial cells were detected after SCI (Additional file 1: Fig. S2A–D). Significant reductions in Caspase-1 and NLRP3 expression in neurons and microglia after SS-31 treatment indicated that SS-31 depressed pyroptosis following SCI. WB assessment was used to determine the expression levels of Caspase-1, GSDMD-N, NLRP3, NLRP1, ASC, IL-1β and IL-18. These pyroptosis-related proteins were substantially more abundant in the SCI group than in the Sham group. SS-31, nevertheless, reduced the expression of all seven proteins (Fig. 2E, F). As a result of these outcomes, SS-31 decreased pyroptosis after SCI.

### SS-31 enhances autophagy after SCI

**Fig. 3:**
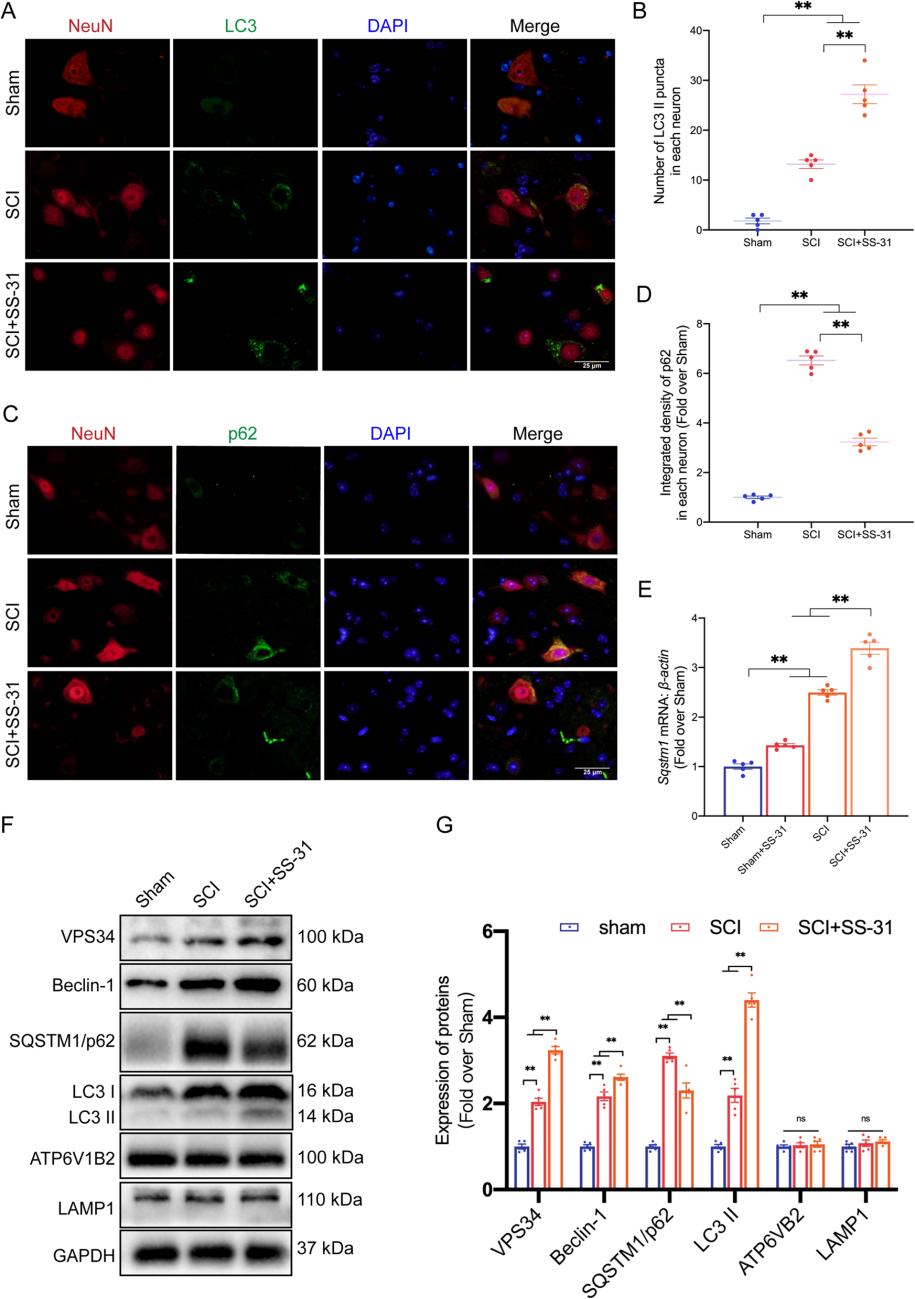
SS-31 enhances autophagy after SCI. **A** Double-IF staining of LC3 and NeuN in the spinal cords of the Sham, SCI, and SCI + SS-31 groups (scale bar = 25 μm). **B** Number of LC3 II puncta in each neuron on the right. **C** Double-IF staining of p62 and NeuN in the spinal cords of the indicated groups (scale bar = 25 μm). **D** Quantified p62 IF data are presented on the right. **E** mRNA level of the *Sqstm1* gene in the impaired spinal cord of the Sham, Sham + SS-31, SCI, SCI + SS-31 groups at 3 dpi. **F** WB for VPS34, Beclin-1, p62, LC3 IIATP6V1B2 and LAMP1 expression levels in the Sham, SCI, and SCI + SS-31 groups. GAPDH was utilized as a loading control. The gels were run under the same experimental conditions, and the cropped blots are shown here. **G** The optical density values of VPS34, Beclin-1, p62, LC3 II, ATP6V1B2 and LAMP1 were quantified and analysed in each group. The data are shown as the mean ± SEM. N = 5. *P < 0.05, **P < 0.01. ns indicates no significance

Increased autophagy has been observed after SCI, and increasing amounts of research have shown that autophagy can function as a prosurvival mechanism by controlling neural cell death to provide neuroprotection [50]. Additionally, previous research has demonstrated that SS-31 promotes autophagy in neurodegenerative diseases [23]. To test the effect of SS-31 on neural autophagy activity after SCI, we examined the protein levels of autophagosomal proteins (Beclin-1, VPS34, and LC3), lysosomal biogenesis-associated biomarkers (ATP6V1B2 and LAMP1), and autophagy substrate proteins (SQSTM1/p62) 3 days after SCI. As shown in Fig. 3A, B, the numbers of LC3 II puncta in each neuron increased following SCI and further increased after additional SS-31 treatment. Furthermore, following treatment with SS-31, the expression of p62 in neurons within the spinal cord lesion was decreased in comparison with that in the SCI group (Fig. 3C, D). As a substrate of autophagy, the SQSTM1/p62 protein is affected not only by autophagic flux, but also by the corresponding gene expression level. Therefore, we detected the mRNA level of *Sqstm1* following SS-31 treatment in both Sham and SCI mice. After SCI, both the mRNA and protein levels of p62 were increased, and the protein level was further increased (Fig. 3E–G), indicating that impairment of autophagic flux occurred after SCI. Moreover, after treatment with SS-31, the mRNA level of *Sqstm1* increased, while the protein level decreased (Fig. 3E–G), which indicated that SS-31 enhanced autophagic flux in SCI mice. Later, we detected the protein levels of autophagy-related genes. WB demonstrated that following SS-31 treatment, the levels of VPS34, Beclin-1, and LC3 II increased, while the level of p62 decreased. Nevertheless, the expression of ATP6V1B2 and LAMP1 did not differ among the three groups (Fig. 3F, G). Overall, these outcomes demonstrated that SCI resulted in impaired autophagy/autophagic flux and that SS-31 alleviated the disruption of autophagic flux.

### The autophagy-activating, pyroptosis-inhibiting and functional recovery-promoting effects of SS-31 are inhibited by CQ

**Fig. 4:**
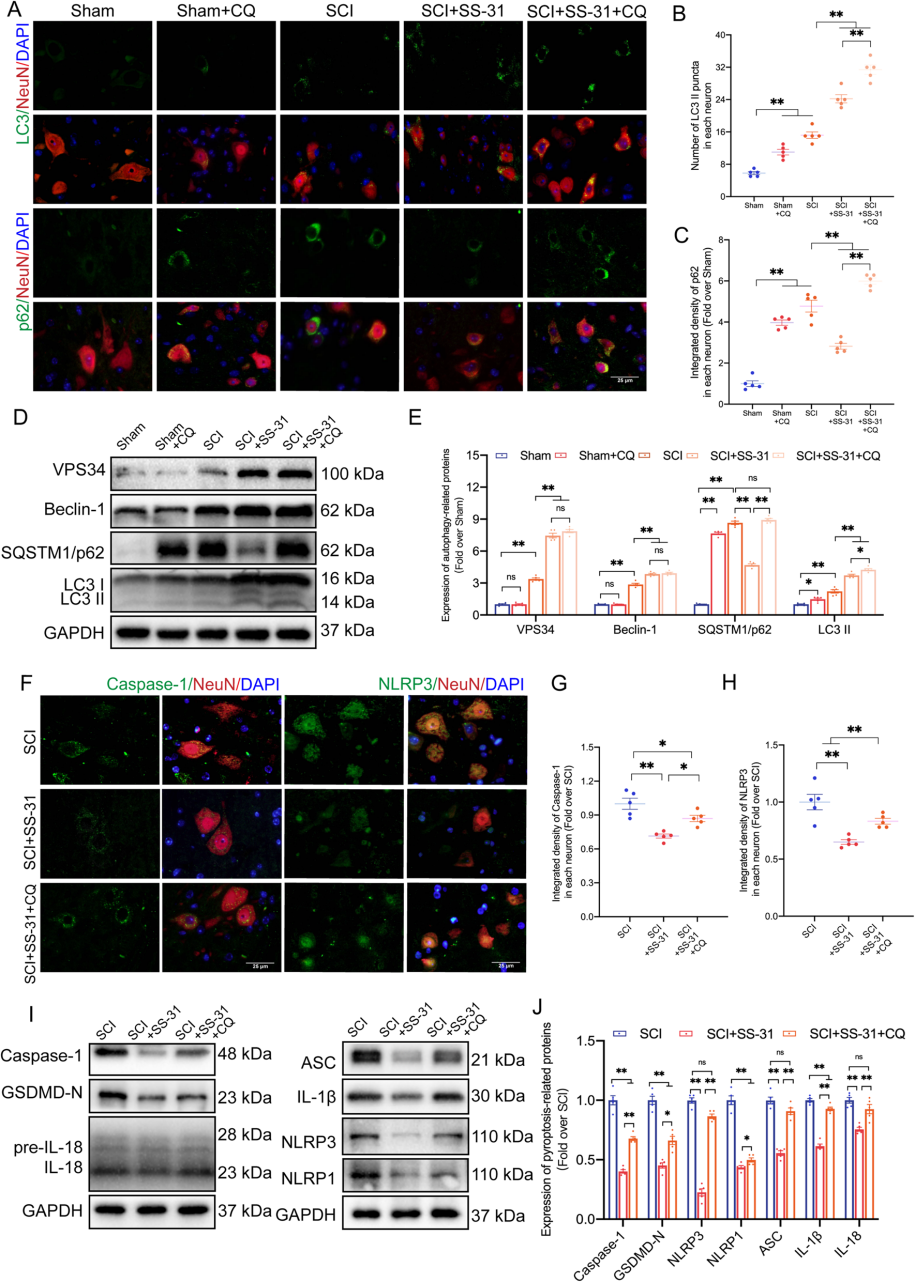
SS-31’s autophagy-activating and pyroptosis-inhibiting effects are inhibited by CQ. **A**–**C** Representative double immunostaining images of LC3/NeuN and p62/NeuN in the injured spinal cord lesions from each group (the Sham, Sham + CQ, SCI, SCI + SS-31, SCI + SS-31 + CQ groups) at 3 dpi (scale bar = 25 μm). The number of LC3 II puncta and the integrated density of p62 in each neuron are shown on the graph. **D** Typical images of WB analyses of VPS34, Beclin-1, p62, and LC3 in the injured spinal cord lesions. GAPDH was utilized as a loading control. **E** Quantified data of the WB results for autophagy-related proteins. **F**–**H** Representative double immunostaining images of Caspase-1/NeuN and NLRP3/NeuN in the injured spinal cord lesions from each group (the SCI, SCI + SS-31, SCI + SS-31 + CQ groups) at 3 dpi (scale bar = 25 μm). The quantitative integrated density of Caspase-1 and NLRP3 in each neuron is shown on the graph. **I** Typical images of WB analyses of Caspase-1, GSDMD-N, NLRP3, NLRP1, ASC, IL-1β and IL-18 in the injured spinal cord lesions. GAPDH was utilized as a loading control. **J** The quantified data of the WB results from pyroptosis-related proteins. The data are shown as the mean ± SEM. (*n* = 5 every group); *P < 0.05, **P < 0.01. ns indicates no significance

To further confirm that the beneficial effects of SS-31 on SCI recovery are due to an increase in autophagic activities, CQ, a classic autophagic flux inhibitor that inhibits lysosomal acidification [51], was used in subsequent research. As the WB and IF results in Fig. 4A–E revealed, the protein levels of VPS34 and Beclin-1 showed no change in the Sham and Sham+CQ groups, while LC3 II and p62 were upregulated in the Sham+CQ group. The results indicated that CQ did not affect the initiation of autophagy, but inhibited the activity of lysosomes and the fusion of lysosomes and autophagosomes, thus inhibiting autophagic flux in non-SCI mice. Then, IF demonstrated that the SCI+SS-31+CQ group had more LC3 II puncta in neurons than the SCI+SS-31 group. IF also showed that p62 levels in neurons were higher in SCI+SS-31+CQ mice than in SCI+SS-31 mice (Fig. 4A–C). The findings indicated that in SCI mice, CQ blocked autophagic flux, which was restored by SS-31. We employed WB to examine autophagy-related proteins in order to determine how CQ affected SS-31 therapy. WB demonstrated that the protein expression of Beclin-1 and VPS34 did not differ significantly between the SCI+SS-31 and SCI+SS-31+CQ groups (Fig. 4D, E). This demonstrated that CQ had no impact on autophagosome recruitment in SCI mice following SS-31 administration. WB also revealed that the LC3 II protein level was increased in the SCI+SS-31+CQ group in comparison with the SCI+SS-31 group. Furthermore, the level of p62 in SCI+SS-31+CQ mice was much greater than that in SCI+SS-31 mice (Fig. 4D, E). All of these investigations indicated that CQ inhibited SS-31’s efficient autophagy enhancement by blocking autophagic flux. Pyroptosis and autophagy are two distinct biological processes that determine cell fates [52]. Thus, we tested whether CQ affected pyroptosis in Sham and SCI mice. As revealed in Additional file 1: Fig. S3A–F, IF and WB showed no differences in the pyroptosis-related proteins of Caspase-1, GSDMD-N, NLRP3, NLRP1, ASC, IL-1β and IL-18 between the Sham and Sham+CQ groups. Our results indicated that although CQ treatment inhibited autophagic flux (Fig. 4A–E), it had no significant effect on pyroptosis in non-SCI mice (Additional file 1: Fig. S3A–F). Later, we examined the expression levels of these pyroptosis-related proteins and tested whether CQ attenuated the inhibition of pyroptosis by SS-31 in SCI mice. As shown in Fig. 4F–H, the integrated density of Caspase-1 and NLRP3 in neurons within the SCI+SS-31+CQ group was larger than that within the SCI+SS-31 group based on the IF results. WB demonstrated that these pyroptosis-related proteins were more abundant in the SCI+SS-31+CQ group than in the SCI+SS-31 group (Fig. 4I, J). These investigations indicated that CQ partly reversed the effects of SS-31 in slowing pyroptosis in SCI.

Next, we considered whether CQ mitigated the impact of SS-31 on the functional recovery of SCI mice. We carried out HE/Masson staining, IF, and motor function evaluation among the SCI, SCI+SS-31, and SCI+SS-31+CQ groups 28 days after SCI. The SCI+SS-31+CQ group had lower MAP2 density and fewer Syn-positive synapses than the SCI+SS-31 group, as shown by IF staining (Additional file 1: Fig. S4A–C). Furthermore, the injured area of the spinal cord in the SCI+SS-31+CQ group exhibited a greater glial scar area than that in the SCI+SS-31 group (Additional file 1: Fig. S4D, E). Next, we performed footprint analysis, inclined plane test and BMS score (Additional file 1: Fig. S4F–J). The results showed that the SCI+SS-31 group had clear restoration of hind leg movement with coordinated crawling on Day 28 following damage, but the SCI+SS-31+CQ group continued to drag their hind legs (Additional file 1: Fig. S4F–H). The inclined plane angle and BMS score were considerably lower within the SCI+SS-31+CQ group at 14, 21, and 28 days post-SCI than within the SCI+SS-31 group (Additional file 1: Fig. S4I, J). These investigations showed that SS-31's capacity to increase autophagy could have been responsible for the improved motor function after SCI.

### SS-31 attenuates LMP and inhibits cPLA2 phosphorylation after SCI

**Fig. 5:**
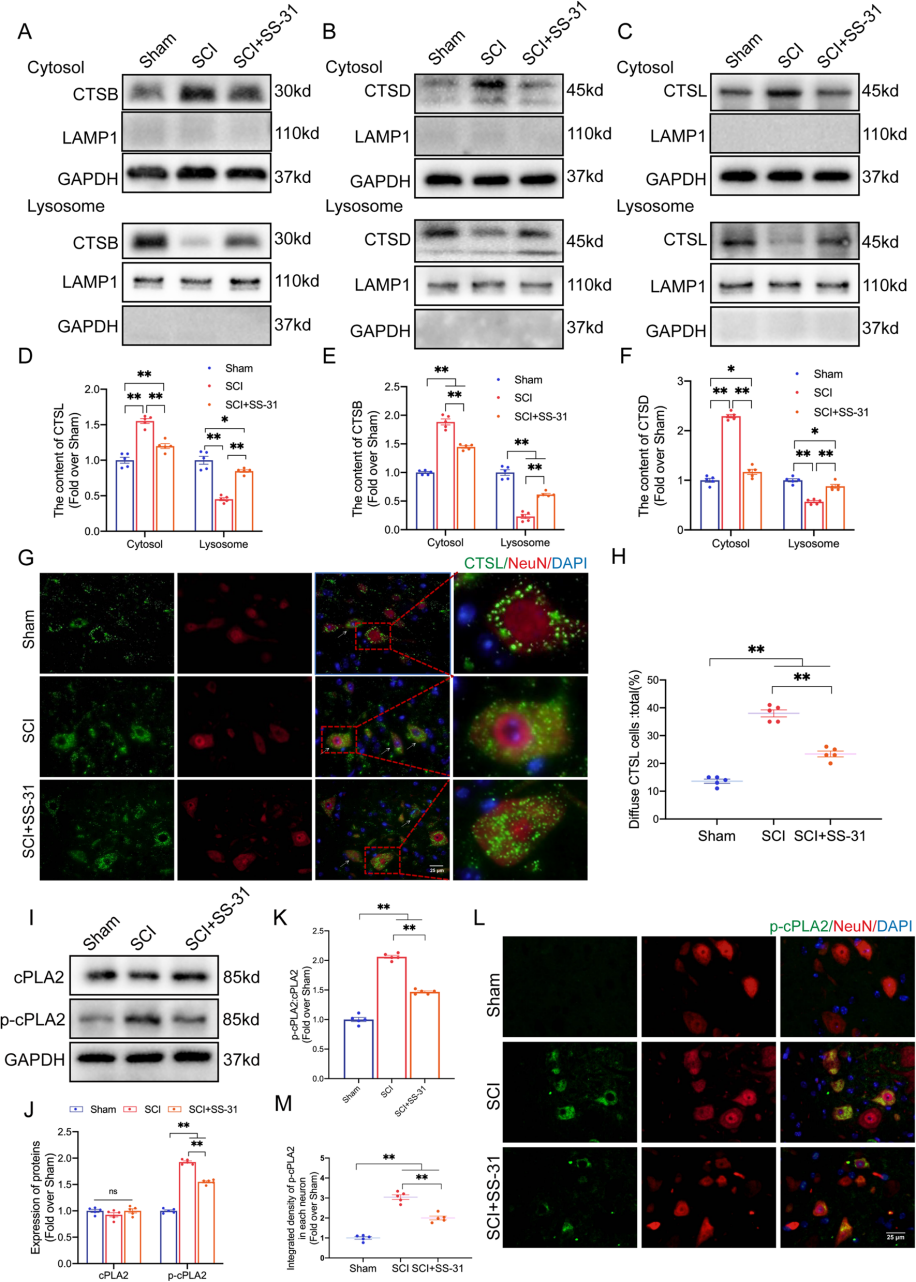
SS-31 attenuates lysosomal membrane permeabilization after SCI. **A**–**C** Protein levels of **A** CTSB, **B** CTSD and **C** CTSL in the cytoplasm and lysosomes extracted from the spinal cords of the Sham, SCI, and SCI + SS-31 groups. The lysosomal membrane protein LAMP1 was used to identify the lysosomal fraction and as a loading control. GAPDH was used to identify the cytosolic fraction and as a loading control. **D**–**F** Quantification of the protein levels of **D** CTSB, **E** CTSD and **F** CTSL in the cytoplasm and lysosomes extracted from the spinal cord. **G** IF staining of NeuN and CTSL in the anterior horns of the spinal cords from the Sham, SCI, and SCI + SS-31 groups on postoperative day 3 (scale bar = 25 μm). The white arrow indicates a neuron with diffuse CTSL. **H** Comparison of the ratios of diffuse CTSL cells in the anterior horn of the spinal cord among the three groups. **I** cPLA2 and p-cPLA2 protein levels in the spinal cords from the three groups on postoperative day 3. **J**–**K** Quantification of the total level, phosphorylated level and ratio of cPLA2 in the spinal cord. **L**–**M** IF staining of NeuN and p-cPLA2 in the anterior horns of the spinal cords from three groups on postoperative day 3 (scale bar = 25 μm). The quantitative integrated density of p-cPLA2 in each neuron is shown on the graph. The data are shown as the mean ± SEM. *n* = 5. *P < 0.05, **P < 0.01. ns indicates no significance

A previous study has described how LMP and the subsequent release of cathepsin B (CTSB) from lysosomes into the cytosol is a signalling pathway that induces pyroptosis [53]. Additionally, mounting evidence suggests that lysosomal damage impairs autophagic flux in neural diseases [54, 55], such as SCI [6], and leads to the accumulation of neuronal autophagosomes. Given the significant impact of SS-31 on pyroptosis inhibition and autophagic flux restoration in our research, we hypothesized that SS-31 may affect lysosome function following SCI. We prepared lysosome-enriched fractions by subcellular fractionation of spinal cord tissue. Then, we directly identified the presence of lysosomal enzymes using WB and IF. The WB results indicated that the SCI group contained more lysosomal enzymes in the cytosolic fractions (including CTSB, CTSD and CTSL) than the Sham group but lower levels of enzymes in the lysosomal fractions (Fig. 5A–F). IF staining of CTSL and NeuN demonstrated that diffuse CTSL cells were more abundant in SCI mice than in Sham mice (white arrows indicate diffuse CTSL in neurons) (Fig. 5G, H). These results suggested that lysosomal enzymes leaked into the cytosol, demonstrating that LMP occurred in SCI. However, treatment with additional SS-31 in SCI mice changed the distribution of lysosomal enzymes (Fig. 5A–H), which suggested that SS-31 attenuated LMP after SCI.

The findings mentioned above demonstrated that under the pathological conditions of SCI, the ability of the lysosomal membrane to act as a barrier was compromised, enabling lysosomal contents to escape into the cytoplasm. According to a previous study, phosphatides are well known to be critical components of neuronal bilayer membranes [56]. Our group has previously reviewed how the activation of cPLA2/Pla2g4a (phospholipase A2, group IVA [cytosolic, calcium-dependent]) produces phosphatide disintegration and membrane breakdown through the hydrolytic action of neuronal membrane phosphatides, resulting in alterations in membrane functions, such as LMP [57]. Considering SS-31’s effect on protecting the lysosomal membrane from permeabilization in SCI, we assumed that it acted by inhibiting the cPLA2 enzyme. Thus, we examined the levels of cPLA2 and p-cPLA2 in the Sham, SCI and SCI+SS-31 groups. The results showed equivalent expression of cPLA2 in the three groups (Fig. 5I–K). Nevertheless, as shown in Fig. 5I–K p-cPLA2 and the ratio of p-cPLA2/cPLA2 were significantly upregulated by SCI, whereas SS-31 attenuated the enhancement of both upregulations. IF showed similar outcomes: the protein level of p-cPLA2 in the SCI group was higher than that in the Sham group, whereas SS-31 downregulated the p-cPLA2 protein level after SCI (Fig. 5L, M). These results indicated that SS-31 effectively inhibited the activation of cPLA2 in neurons after SCI. Moreover, we performed IF experiments to assess the specific expression of p-cPLA2 in other cell types, including microglia, astrocytes and oligodendrocytes (Additional file 1: Fig. S5A–F). The results showed that the number of p-cPLA2-positive microglia and oligodendrocytes were increased in SCI mice but SS-31 treatment attenuated this upregulation. However, the number of p-cPLA2-positive astrocytes showed no significant differences in the Sham, SCI and SCI+SS-31 groups.

Additionally, the enzymatic activity assay demonstrated that cPLA2 activity increased following SCI and decreased following the administration of SS-31 (Additional file 1: Fig. S6A). Later, we performed subcellular fractionation of spinal cord tissue and prepared lysosome-enriched and cytosolic fractions. ELISA was employed to determine the activity of CTSD and NAGLU in lysosomes. As shown in Additional file 1: Fig. S6B, C, the activity levels of both lysosomal enzymes were lower in the SCI group than in the Sham group. Furthermore, the enzymes were significantly more active within the SCI group's cytosolic fractions than within the Sham group (Additional file 1: Fig. S6D, E). These SCI-induced alterations were significantly attenuated by SS-31 (Additional file 1: Fig. S6B–E), suggesting restoration of lysosomal function. All these results showed that SS-31 inhibited cPLA2 from being phosphorylated and decreased the damage to lysosomal membranes after SCI.

### SS-31 attenuates LMP and pyroptosis and enhances autophagy by suppressing the activation of cPLA2 after SCI

**Fig. 6:**
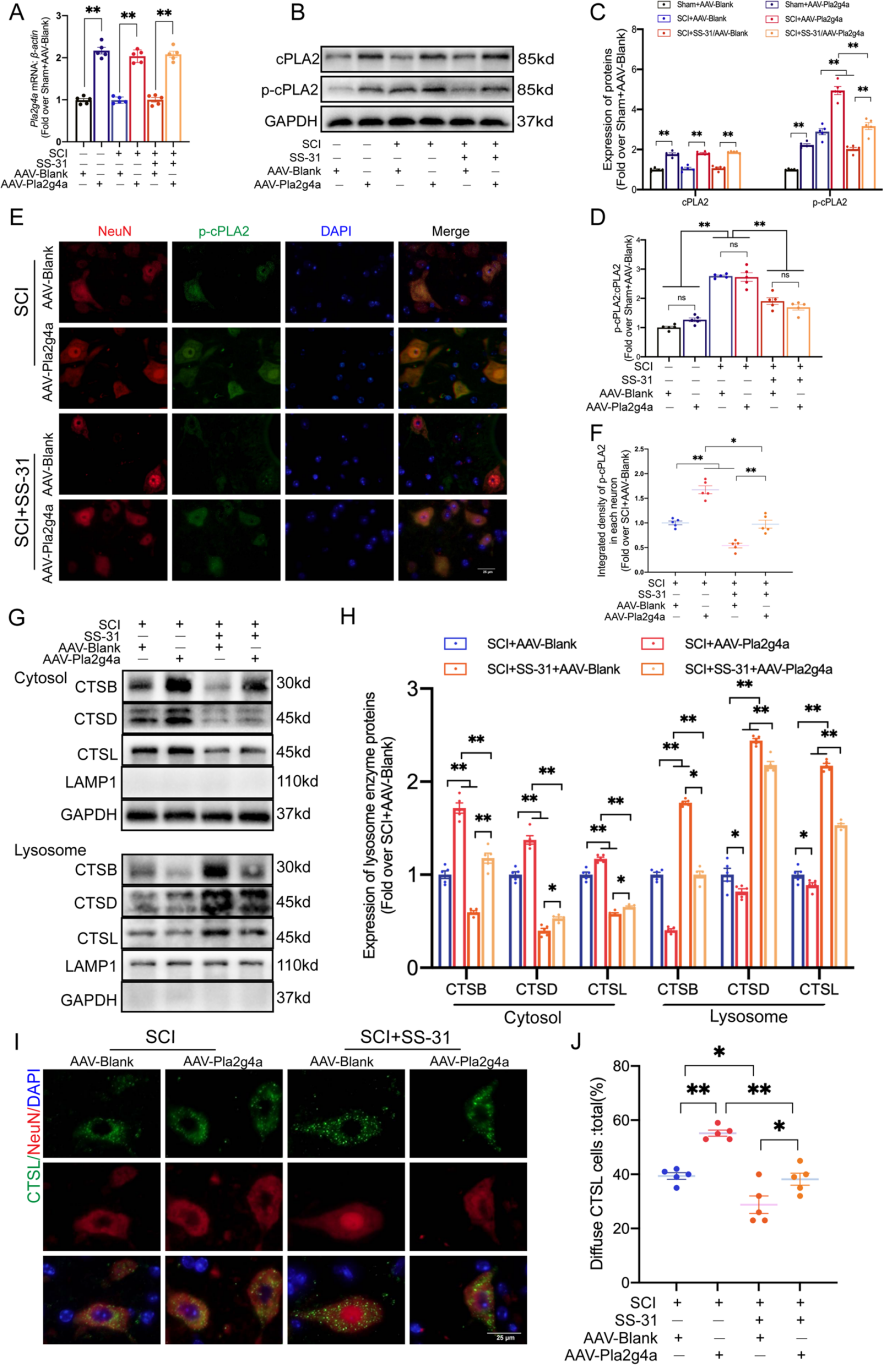
SS-31 inhibits LMP by downregulating cPLA2. **A** mRNA level of *Pla2g4a* gene in the impaired spinal cords of the Sham + AAV-Blank, Sham + AAV-Pla2g4a, SCI + AAV-Blank, SCI + AAV-Pla2g4a, SCI + SS-31 + AAV-Blank, and SCI + SS-31 + AAV-Pla2g4a groups at 3 dpi. **B**–**D** WB analysis of cPLA2 and p-cPLA2 expression in the injured spinal cord lesions in the six groups. The protein levels of p-cPLA2 and total cPLA2 and the ratio were quantified in the injured spinal cord. **E**–**F** Representative IF images of p-cPLA2 and NeuN in the impaired spinal cord areas from the SCI + AAV-Blank, SCI + AAV-Pla2g4a, SCI + SS-31 + AAV-Blank, and SCI + SS-31 + AAV-Pla2g4a groups at 3 dpi (scale bar = 25 μm). The quantification data are shown on the right. **G** CTSB, CTSD, and CTSL protein concentrations were extracted in the cytoplasm and lysosomes from the spinal cords of the indicated four groups. **H** Quantification of the protein levels of CTSB, CTSD and CTSL in the cytoplasm and lysosomes extracted from the spinal cord. **I** IF staining of NeuN and CTSL in the anterior horns of the spinal cords from the SCI + AAV-Blank, SCI + AAV-Pla2g4a, SCI + SS-31 + AAV-Blank, and SCI + SS-31 + AAV-Pla2g4a groups on postoperative day 3 (scale bar = 25 μm). **J** Comparison of the ratio of diffuse CTSL cells in the anterior horn of the spinal cord among the four groups. The data are shown as the mean ± SEM. *n* = 5. *P < 0.05, **P < 0.01. ns indicates no significance

To learn more about the function of SS-31-mediated cPLA2 inhibition in attenuating LMP, we employed AAV-Pla2g4a to activate cPLA2 and devised a rescue experiment to compare the following 6 groups: Sham+AAV-Blank, Sham+AAV-Pla2g4a, SCI+AAV-Blank, SCI+AAV-Pla2g4a, SCI+SS-31+AAV-Blank and SCI+SS-31+AAV-Pla2g4a groups. qPCR and WB showed that the application of AAV-Pla2g4a significantly upregulated *Pla2g4a* mRNA and cPLA2/p-cPLA2 protein expression but did not change the ratio of p-cPLA2 to cPLA2 in the Sham, SCI and SCI+SS-31 groups (Fig. 6A–D). IF staining demonstrated that the protein levels of p-cPLA2 in neurons within the SCI+SS-31+AAV-Pla2g4a group were consistently higher than those within the SCI+SS-31+AAV-Blank group (Fig. 6E, F). All of these findings demonstrated that AAV-Pla2g4a transfection activated cPLA2.

**Fig. 7:**
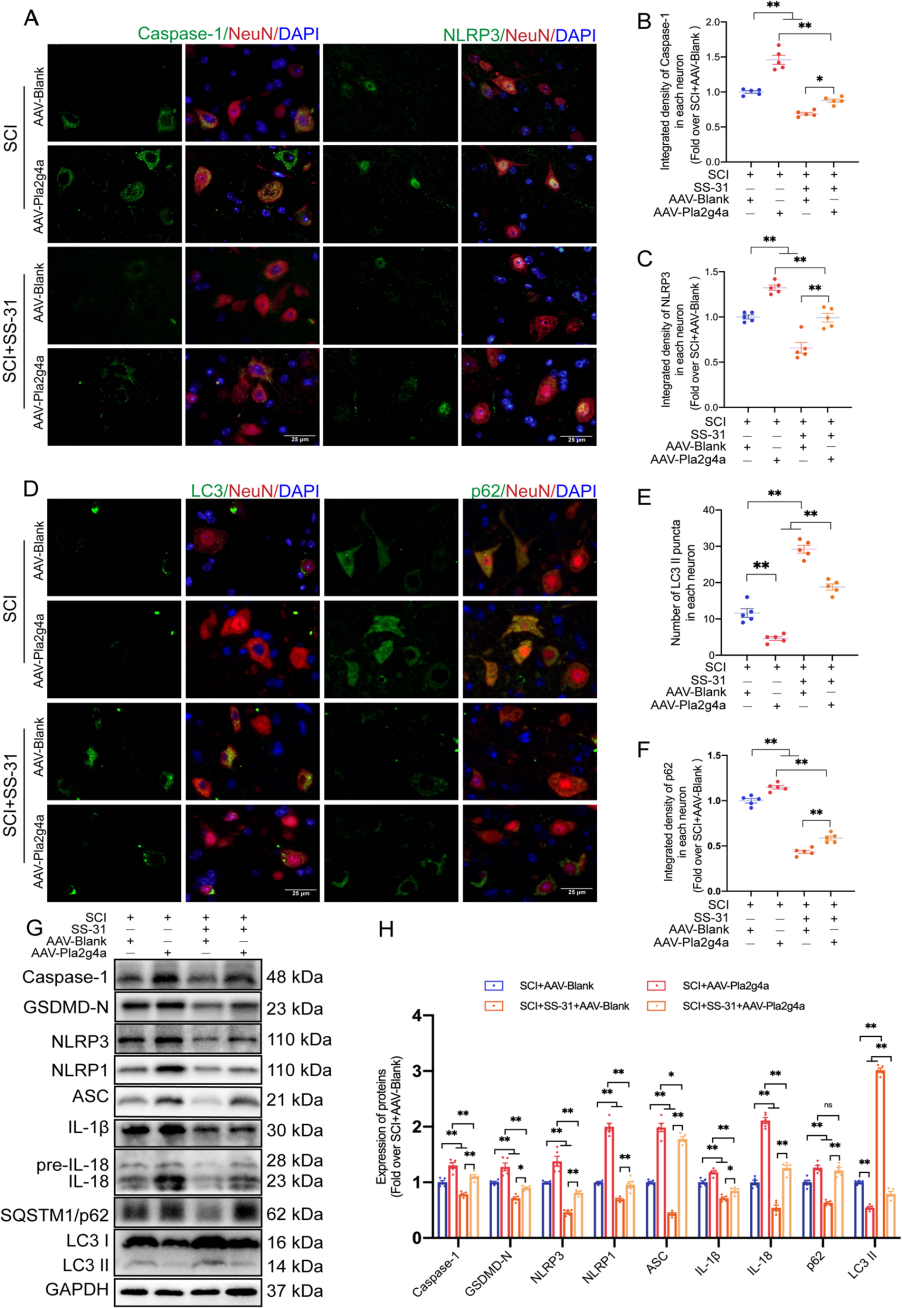
SS-31 inhibits pyroptosis and enhances autophagy by downregulating cPLA2. **A**–**C** Representative double-IF images for Caspase-1/NeuN and NLRP3/NeuN in spinal cord lesions from all groups (SCI + AAV-Blank, SCI + AAV-Pla2g4a, SCI + SS-31 + AAV-Blank, SCI + SS-31 + AAV-Pla2g4a groups) at 3 dpi (scale bar = 25 μm). The quantitative integrated density of Caspase-1 and NLRP3 in each neuron is shown on the graph. **D**–**F** Representative double-IF images for LC3/NeuN and p62/NeuN in spinal cord lesions from all groups (the SCI + AAV-Blank, SCI + AAV-Pla2g4a, SCI + SS-31 + AAV-Blank, SCI + SS-31 + AAV-Pla2g4a groups) at 3 dpi (scale bar = 25 μm). The number of LC3 II puncta and the quantitative integrated density of p62 in each neuron is shown on the graph. **G** Typical images of WB analyses of Caspase-1, GSDMD-N, NLRP3, NLRP1, ASC, IL-1β, IL-18, p62, and LC3 II in the impaired spinal cord. GAPDH was utilized as a loading control. **H** Quantification results of the related protein levels in the indicated groups. The data are shown as the mean ± SEM. *n* = 5. *P < 0.05, **P < 0.01. ns indicates no significance

Our team then explored whether LMP is influenced by SS-31's impact on cPLA2. As shown in the WB results in Fig. 6G, H, compared to the SCI+SS-31+AAV-Blank group, the SCI+SS-31+AAV-Pla2g4a group showed a remarkable tendency toward leakage of lysosomal enzymes from lysosomes into the cytoplasm. Based on the IF data (Fig. 6I, J), significantly more diffuse CTSL cells were present in SCI+SS-31+AAV-Pla2g4a mice than in SCI+SS-31+AAV-Blank mice. Recent research has demonstrated that inhibiting cPLA2 not only decreases LMP, but also restores autophagic flux and is connected to reduced neuronal cell damage [6, 19]. In addition, a review focusing on pyroptosis in CNS trauma has proposed that inhibiting cPLA2 may attenuate pyroptosis [53]. Considering the strong ability of SS-31 in the inhibition of cPLA2, we hypothesized that it is responsible for regulating pyroptosis and autophagy. IF staining showed that the integrated density of NLRP3 and Caspase-1 in neurons was significantly lower in the SCI+SS-31+AAV-Blank group than in the SCI+AAV-Blank group, but the opposite results were observed in the SCI+SS-31+AAV-Pla2g4a group (Fig. 7A–C). WB produced a similar outcome: the levels of the pyroptosis-related proteins Caspase-1, GSDMD-N, NLRP3, NLRP1, ASC, IL-1β, and IL-18 were lower in the SCI+SS-31+AAV-Blank group than in the SCI+AAV-Blank group; however, they were elevated in the SCI+SS-31+AAV-Pla2g4a group (Fig. 7G, H). As shown in Fig. 7D–H, the results of IF staining and WB demonstrated that the numbers of LC3 II puncta and the expression of LC3 II were higher in the SCI+SS-31+AAV-Blank group than in the SCI+AAV-Blank group and were significantly lower relative to the SCI+SS-31+AAV-Pla2g4a group. Compared to those in the SCI+AAV-Blank group, the levels of p62 protein according to IF and WB were decreased in the SCI+SS-31+AAV-Blank group and increased in the SCI+SS-31+AAV-Pla2g4a group (Fig. 7D–H). We also carried out experiments on the Sham, Sham+AAV-Blank and Sham+AAV-Pla2g4a groups to test whether overexpressing *Pla2g4a* affected autophagy, pyroptosis and LMP in non-SCI mice (Additional file 1: Fig. S7A–N). Our results showed that AAV-Pla2g4a injection upregulated the expression of cPLA2 in Sham mice but did not significantly affect autophagy, pyroptosis or LMP (Additional file 1: Fig. S7A–N). In summary, our outcomes demonstrate that SS-31 therapy suppresses cPLA2, which is a crucial approach by which SS-31 slows LMP, inhibits pyroptosis, and promotes autophagy in SCI mice.

Finally, we explored the therapeutic impact of SS-31 following transfection with AAV-Pla2g4a to determine whether cPLA2 is responsible for an improvement in motor function after SCI with SS-31 treatment. As expected, the SCI+SS-31+AAV-Pla2g4a group showed fewer Syn-positive synapses, decreased MAP2 expression in ventral motor neurons (Additional file 1: Fig. S8A, B, D), and a larger area of glial scarring than the SCI+SS-31+AAV-Blank group (Additional file 1: Fig. S8C, E). Footprint analyses demonstrated that the SCI+SS-31+AAV-Pla2g4a mice moved less within their rear legs and exhibited more toe dragging while crawling together than the SCI+SS-31+AAV-Blank mice (Additional file 1: Fig. S8F–H). Furthermore, compared with the SCI+SS-31+AAV-Blank group, the SCI+SS-31+AAV-Pla2g4a mice exhibited a substantially lower BMS score and inclined plane test result at 21 and 28 days following SCI (Additional file 1: Fig. S8I, J). These findings indicate that SS-31's beneficial therapeutic benefits are mediated by enhancement of autophagy, which inhibits pyroptosis as well as LMP by depressing activation of cPLA2.

### SS-31 inhibits cPLA2 through the p38-MAPK signalling pathway

**Fig. 8:**
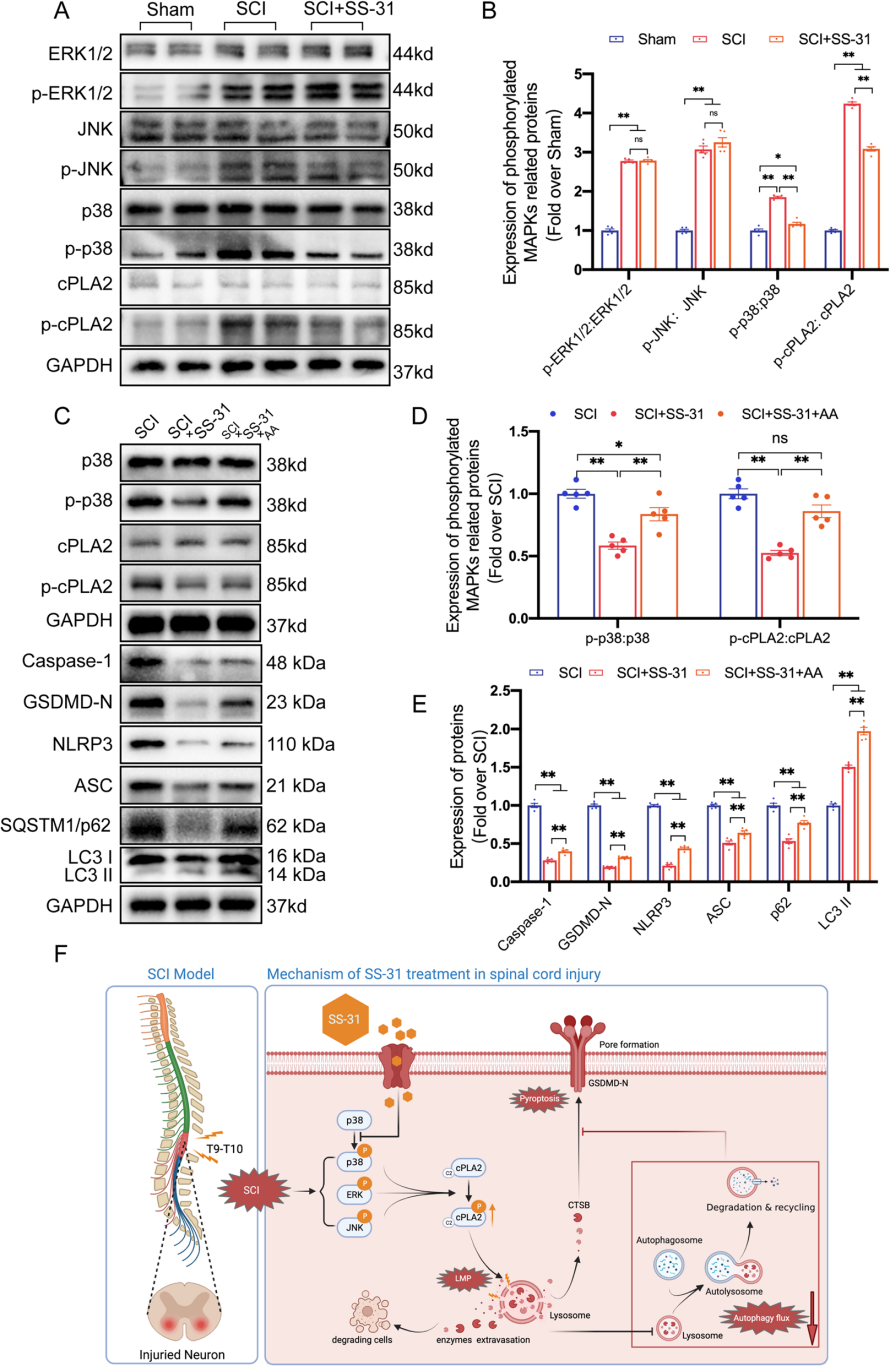
SS-31 inhibits cPLA2 through the MAPK-p38 signalling pathway. **A** Typical images of WB results of ERK1/2, p-ERK1/2, JNK, p-JNK, p38, p-p38, cPLA2, and p-cPLA2 expression in the injured spinal cord lesions in Sham, SCI and SCI + SS-31 groups. GAPDH was utilized as a loading control. **B** Quantification of the protein levels of ERK1/2, p-ERK1/2, JNK, p-JNK, p38, p-p38, cPLA2, and p-cPLA2. **C** Typical images of WB results of p38, p-p38, cPLA2, p-cPLA2, Caspase-1, GSDMD-N, NLRP3, ASC, p62 and LC3 expression in the injured spinal cord lesions in the SCI, SCI + SS-31, and SCI + SS-31 + AA groups. GAPDH was utilized as a loading control. **D** Quantification of the protein levels of p38, p-p38, cPLA2 and p-cPLA2. **E** Quantification of the autophagy- and pyroptosis-related protein levels in the indicated groups. **F** Schematic diagram showing the potential protective effect of SS-31 against SCI. The data are shown as the mean ± SEM. *n* = 5. *P < 0.05, **P < 0.01. ns indicates no significance

Examination of the whole sequence of cPLA2 clearly reveals that this enzyme contains a consensus phosphorylation motif (involving Ser-505) that is a target of the MAPK family, which consists of three major components: ERK, p38, and JNK [58]. In addition, many studies have demonstrated that SS-31 shows great power in inhibiting the activation of the p38 MAPK signalling pathway in a variety of ischaemia‒reperfusion injury disease models [31, 59, 60]. Therefore, we performed further research to learn more about the relationship between SS-31 and the MAPK signalling pathway in SCI. As shown in Fig. 8A, B, p38, ERK1/2, JNK, and cPLA2 were all significantly activated after SCI. The administration of SS-31 reduced the ratio of p-cPLA2/cPLA2 and p-p38/p38, while the ratio of p-ERK1/2/ERK1/2 and p-JNK/JNK did not differ in the SCI+SS-31 group from those in the SCI group. These results suggest that SS-31 inhibits the p38-MAPK pathway rather than the ERK1/2- or JNK-MAPK pathway.

To determine whether the p38-MAPK signalling pathway affected SS-31's impact on cPLA2, we focused on the effects of AA (a known p38 agonist [40]) on the MAPK signalling pathway. The investigations demonstrated that the mice in the SCI+SS-31+AA group had higher p-p38/p38 and p-cPLA2/cPLA2 ratios than those in the SCI+SS-31 group, indicating the efficacy of AA (Fig. 8C, D). As shown in Fig. 8C–E, when compared to the SCI+SS-31 group, Caspase-1, GSDMD-N, NLRP3, ASC, p62, and LC3 II were upregulated in the SCI+SS-31+AA group, demonstrating that AA effectively reversed the SS-31-mediated restoration of autophagic flux and inhibition of pyroptosis in the SCI+SS-31 group. In addition, ELISA demonstrated that the enzymes CTSD and NAGLU were significantly less active within lysosomes in the SCI+SS-31+AA group than in the SCI+SS-31 group. Furthermore, the cytosolic fractions from the SCI+SS-31+AA group had substantially higher activity of both enzymes than those from the SCI+SS-31 group (Additional file 1: Fig. S9A–D). In short, our outcomes show that SS-31 inhibits cPLA2 from becoming active in SCI by blocking the p38-MAPK signalling pathway.

## Discussion

As a consequence of traumatic spinal cord injury, irreversible nerve damage occurs [1]. Depending on the pathological process, traumatic spinal cord injury is classified into 2 stages: primary injury and secondary injury. Thus far, abundant research has focused on the secondary injury stage, in which neuroinflammation and programmed cell death are the main mechanisms. Anti-inflammatory, antipyroptotic and neuroprotective effects of SS-31 have been demonstrated in many neurodegenerative diseases [26, 27]. However, investigations on the application of SS-31 for SCI have never been reported. In the present work, we found that damaged spinal cord induced LMP, impaired autophagic flux, and activated pyroptosis, which resulted in neuronal injury and a deficiency in the recovery of motor function. Notably, inhibition of the p38-cPLA2 signalling pathway with the aromatic cationic peptide SS-31 alleviated these defects. SS-31 may therefore be useful as a therapeutic agent for SCI in the future.

Pyroptosis is a recently identified mode of regulated programmed cell death accompanied by inflammation [53]. During pyroptosis, the inflammasome is activated, membrane holes develop, the membrane swells and breaks, and the intracellular contents are released [53]. Precise regulation of pyroptosis may prevent excessive neuronal death. Therefore, we utilized established molecular approaches to determine whether SS-31 prevented pyroptosis. The outcomes demonstrated that SS-31 substantially reduced the expression levels of proteins associated with pyroptosis following SCI. Our group has previously explored the involvement of autophagy in SCI [7, 61, 62]. Basal levels of autophagy are vital for maintaining cell homeostasis and are required for neural cell function and survival. According to current research, autophagic flux following CNS damage may increase or decrease depending on the site and extent of the injury [50]. As a result, autophagy may function in either a positive or negative manner following an injury. Nevertheless, it seems that restoring and/or enhancing autophagic flux may enhance cell survival and accelerate functional recovery following damage in all circumstances [63, 64]. This implies that the autophagy pathway could be a promising treatment target for SCI. The present study examined autophagic flux and autophagy-related protein levels following SCI with and without SS-31 therapy. These investigations demonstrated that SS-31 increased autophagic flux in SCI. Previous research has demonstrated that activating autophagy in the context of CNS trauma inhibits the pyroptotic death of cells [7, 61, 65]. The results of our experiments corroborated these observations. When autophagic flux was blocked, the levels of pyroptosis increased dramatically. This finding indicates that SS-31, by activating autophagic flux, may minimize pyroptotic death of cells in SCI. Notably, the impacts of CQ on autophagic flux did not entirely cancel out the impacts of SS-31 on pyroptosis. Based on our observations, SS-31 may prevent pyroptosis through another distinct mechanism. However, the potential mechanism needs to be further explored. To summarize, our outcomes demonstrate that SS-31 reduces pyroptosis partially by promoting the autophagic flux process, which is beneficial in alleviating SCI.

LMP, an important type of lysosomal damage, is caused by reactive oxygen species (ROS), intralysosomal Fenton reactions and other cell stresses [66]. Researchers have recently started to focus on the role of LMP in CNS trauma [6, 19, 67]. Abundant evidence has shown that lysosomal damage results in neuronal autophagosome accumulation and inhibits autophagic flux in neural diseases [54, 55]. Nevertheless, no drug has been found to ameliorate SCI by inhibiting LMP. Considering the previous results that SS-31 restored autophagic flux after SCI, we hypothesized that SS-31 may interfere with the process of autophagy by affecting LMP. Our findings validated our hypothesis: SS-31 prevented cathepsins from migrating from the lysosome to the cytoplasm and restored lysosomal enzyme function. Overall, our research is the first to develop a drug (SS-31) that attenuates SCI partially by inhibiting LMP.

To clarify the potential mechanism underlying the therapeutic effect of SS-31 on SCI, we explored probable upstream processes linked to pyroptosis, autophagy, and LMP. It was recently reported that cPLA2, a phospholipase family member, is required for modulation of autophagy/lysosomal biogenesis and pyroptosis in traumatic CNS injuries [6, 53, 57]. As the primary mediator of neuroinflammation, cPLA2 may break down membrane phosphatides at the sn-2 position to produce lysophospholipids and ω3-polyunsaturated fatty acids, the majority of which are arachidonic acid derivatives and cause further damage [54]. The lysosomal membrane can also be hydrolysed by cPLA2 under pathological conditions, subsequently leading to LMP. Since cPLA2 can influence lysosomal formation, lysosomal dysfunction caused by cPLA2 impedes the autophagic process and contributes to cell death. In addition, a recent review discussed how cPLA2-induced LMP resulting in the leakage of CTSB could be an important way to activate pyroptosis [53]. The function of cPLA2 was therefore explored in this research. In our study, we discovered that SS-31 inhibited the activation of cPLA2 in neurons, microglia and oligodendrocytes after SCI. Moreover, inhibiting the phosphorylation of cPLA2 with SS-31 enhanced the expression of proteins associated with autophagy, reduced the expression of proteins associated with pyroptosis, and prevented LMP [68]. These outcomes show that SS-31 increases autophagy, decreases pyroptosis, and attenuates LMP by inhibiting the activation of cPLA2.

Next, we further investigated how SS-31 regulates cPLA2. The entire sequence of cPLA2 has a consensus phosphorylation motif (containing Ser-505) that targets the MAPK family (including the p38, ERK1/2 and JNK signalling pathways) [69]. All three of these pathways are involved in the generation of cytokines and mediators that promote inflammation in the presence of SCI [57]. In prior work, intrathecal administration of SB203580, a selective p38-MAPK inhibitor, halted the expression of p-cPLA2 and p-p38 and had a neuroprotective effect after transient focal cerebral ischaemia [70]. In addition, many studies have demonstrated that SS-31 mainly inhibits p38-MAPK signalling in hypertensive cardiomyopathy [60], diabetic nephropathy [59] and doxorubicin‑induced cardiotoxicity [31]. Here, we had strong reasons to speculate that SS-31 may exert its strong protective effects against SCI in a manner dependent on the p38-cPLA2-MAPK signalling pathway. In our study, the activation of p38 and cPLA2 after SCI was attenuated by SS-31. In addition, we used a p38 agonist (AA) to upregulate the expression of phosphorylated p38. To summarize, we here provide the first report that SS-31 inhibits the activation of cPLA2 in SCI via the p38-MAPK signalling pathway.

The fact that SS-31 has a high therapeutic impact suggests that it has much promise for use in clinical settings. However, more research on the pharmacological mechanisms of SS-31 is required before it can be utilized within the clinic. (1) Because SS-31 is a peptide that targets mitochondria, it may concentrate over 1000 times within the inner membrane of mitochondria regardless of the membrane potential. Previous research has demonstrated that SS-31 preserves mitochondrial function by reducing the generation of ROS [71], which are also significant contributors to secondary damage in SCI. Thus, more research on the impact of SS-31 on ROS in SCI is needed. (2) The production of many metabolites is induced by cPLA2, including arachidonic acid, prostaglandin E2, and lysophosphatidic acid, which can also meditate neuroinflammation [72]. It remains unknown whether SS-31 exerts its anti-inflammatory effect by inhibiting the downstream products of cPLA2; subsequent experiments should focus on this possibility. (3) Our results show that SS-31 inhibits the p38 pathway rather than the JNK or ERK1/2 MAPK signalling pathway. Nevertheless, previous studies have shown that once CNS trauma occurs, the three signalling pathways do not function independently; rather, they collaborate to affect the phosphorylation of cPLA2 [57, 73]. According to Kishimoto's research [74], the cPLA2^+/+^ ischaemic cortex activates p38, ERK1/2, and JNK1/2 to a greater extent than the cPLA2^–/–^ischaemic cortex 6 h after reinfusion. The author thought the outcomes might result in a positive feedback loop in which cPLA2-dependent oxidative stress increases kinase stimulation, which leads to increased cPLA2 stimulation. Thus, combined treatment with SS-31 and other specific JNK or ERK1/2 inhibitors may interrupt the positive feedback loop of cPLA2 stimulation and produce additive or synergistic impacts in SCI. (4) The oral route is a drug delivery route with high safety and good compliance and has thus gradually become the main route of administration. Nevertheless, the structure and physiological function of the gastrointestinal tract make the bioavailability and half-life of peptide drugs low after oral administration. In previous studies, SS-31 has usually been administered by injection. In the future, SS-31 might be mixed with hydrogels and administered to the injured area of the spinal cord in a direct manner. This would enable sustained release and prolonged efficacy of the medicine.

## Conclusion

Our research shows that SS-31 inhibits the phosphorylation of cPLA2 by restraining the p38-MAPK signalling pathway. The reduction in p-cPLA2 restores autophagic flux and attenuates pyroptosis and LMP. Ultimately, these beneficial effects of SS-31 result in better outcomes in SCI. Figure 8F shows a schematic depiction of our study. Overall, these findings provide new preclinical evidence for SS-31's therapeutic impact on SCI. Future studies to advance the clinical translation of SS-31 as a therapy for SCI patients are eagerly anticipated.

## Supplementary Information

**Additional file 1: Figure S1. ** SS-31 does not affect the histological morphology and motor function in non-SCI mice. (A) Photographs of spinal cord sections in the Sham and Sham+SS-31 groups stained with antibody MAP2 (green) (scale bar = 25 μm). (B) MAP2 optical density within uninjured spinal cord on day 28. (C) Photographs of spinal cord sections in the respective groups stained with antibody Syn (green)/NeuN (red) (scale bar = 20 μm). (D) Relevant quantitative results for motor neuron-contacting synapse amounts on day 28 after sham surgery. (E) Photographs of mouse footprints on day 28 following sham surgery. Blue: forepaw print; Red: hind paw print. (F, G) Toe dragging (%) and stride length (cm) analyses of mice at 28 days after sham surgery. (H) Longitudinal spinal cord sections from the groups at 28 days after sham surgery were examined via HE dyeing and Masson dyeing (scale bar = 1000 μm). (I) Basso mouse scale (BMS) for the indicated groups at days 0, 1, 3, 7, 14, 21, and 28. The data are shown as the mean ± SEM. n = 5. *P < 0.05, **P < 0.01. ns indicates no significance. **Figure S2.** SS-31 attenuates pyroptosis in microglia after SCI. (A) Typical images of immunofluorescence staining for Caspase-1 and microglia colocalization and in the spinal cords of the Sham, SCI, and SCI+SS-31 groups (scale bar= 25μm) (B) Quantified Caspase-1 immunofluorescence data are presented on the right. (C) Typical images of immunofluorescence staining for NLRP3 and microglia colocalization and in the spinal cords of the Sham, SCI, and SCI+SS-31 groups (scale bar= 25μm) (D) Quantified NLRP3 immunofluorescence data are presented on the right. The white arrows indicate Caspase-1 or NLRP3-positive microglia. The data are shown as the mean ± SEM. n = 5. *P < 0.05, **P < 0.01. ns indicates no significance. **Figure S3.** CQ treatment does not affect pyroptosis in non-SCI mice. (A-D) Representative double immunofluorescence images for Caspase-1/NeuN and NLRP3/NeuN in spinal cord from Sham and Sham+CQ groups at 3 dpi (scale bar= 25μm). The quantitative integrated densities of Caspase-1 and NLRP3 in each neuron are shown on the graph. (E) Typical images of WB results of Caspase-1, GSDMD-N, NLRP3, NLRP1, ASC, IL-1β, IL-18 in the spinal cord. GAPDH was utilized as a loading control. (F) Quantification results of the pyroptosis-related protein levels from (E) in both groups. The data are shown as the mean ± SEM. ns indicates no significance. **Figure S4. **SS-31’s functional recovery effect is inhibited by CQ. (A) Photographs of spinal cord sections in the SCI, SCI+SS-31, SCI+SS-31+CQ groups stained with antibody MAP2 (green) (scale bar = 25 μm). Photographs of spinal cord sections in the respective groups stained with Syn (green)/NeuN (red) (scale bar = 20 μm). (B) Relevant quantitative results for motor neuron-contacting synapse amounts on day 28 after SCI. (C) MAP2 optical density within a spinal cord subjected to injury on day 28. (D) Longitudinal spinal cord sections at 28 dpi after SCI stained with HE dyeing and Masson dyeing (scale bar = 1000 μm). (E) The lesion area in injured spinal cord measured from Masson staining. (F) Photographs of mouse footprints on day 28 following spinal cord injury. Blue: forepaw print; Red: hind paw print. (G, H) Toe dragging (%) and stride length (cm) analyses of mice at 28 dpi after SCI. (I, J) Inclined plane test and Basso mouse scale (BMS) for the indicated groups at days 0, 1, 3, 7, 14, 21, and 28. The data are shown as the mean ± SEM. n = 5. *P < 0.05, **P < 0.01. ns indicates no significance. **Figure S5.** SS-31 affects the p-cPLA2 expression in glial cells. (A-B) Typical images of immunofluorescence staining for p-cPLA2 and microglia colocalization in the spinal cords of the Sham, SCI, and SCI+SS-31 groups. The white arrow indicates the p-cPLA2 positive microglia. The ratio of p-cPLA2 positive cells to the total is shown on the right. (C-D) Typical images of immunofluorescence staining for p-cPLA2 and oligodendrocyte colocalization in the spinal cords of three groups. The white arrow indicates the p-cPLA2 positive oligodendrocyte. The ratio of p-cPLA2 positive cells to the total is shown on the right. (E-F) Typical images of immunofluorescence staining for p-cPLA2 and astrocyte colocalization in the spinal cords of three groups. The white arrow indicates the p-cPLA2 positive astrocyte. The ratio of p-cPLA2 positive cells to the total is shown on the right. The data are shown as the mean ± SEM. n = 5. *P < 0.05, **P < 0.01. ns indicates no significance. **Figure S6. **SS-31 inhibits the activity of cPLA2 and protect the lysosomal enzymes activity in lysosomes. (A) ELISA results showing the activity of cPLA2 in the spinal cord from Sham, SCI, and SCI+SS-31 groups on postoperative day 3. (B-D) ELISA results of the activity of lysosomal enzymes CTSD and NAGLU in lysosomal (B, C) and cytosolic (D, E) fractions extracted from the spinal cord of Sham, SCI, and SCI+SS-31 groups on postoperative day 3. The data are shown as the mean ± SEM. n = 5. *P < 0.05, **P < 0.01. ns indicates no significance. **Figure S7. **Pla2g4a overexpression doesn’t affect pyroptosis, autophagy or LMP in non-SCI mice. (A-B) Representative immunofluorescence images of p-cPLA2 and NeuN in the anterior horn areas of spinal cord from Sham, Sham+AAV-Blank, Sham+AAV-Pla2g4a groups after 3 dpi. (scale bar= 25μm). The quantification data were shown on the right. (C) cPLA2 and p-cPLA2 protein levels in the spinal cord from three groups on postoperative day 3. (D-F) Representative double immunofluorescence images for NLRP3/NeuN and Caspase-1/NeuN in spinal cord from three groups at 3 dpi (scale bar= 25μm). The quantitative integrated densities of NLRP3 and Caspase-1 in each neuron are shown on the graph. (G) Typical images of WB analyses of Caspase-1, GSDMD-N, NLRP3, NLRP1, ASC in the spinal cord. GAPDH was utilized as a loading control. (H-J) Representative double immunofluorescence images for p62/NeuN and LC3/NeuN in spinal cord lesions from three groups at 3 dpi (scale bar= 25μm). The number of LC3 II puncta and the quantitative integrated density of p62 in each neuron are shown on the graph. (K) Typical images of WB analyses of p62 and LC3 in the spinal cord. GAPDH was utilized as a loading control. (L) Comparison of the ratio of diffuse CTSL cells in the anterior horn of spinal cord among three groups. (M) Immunofluorescence staining of NeuN and CTSL in the anterior horn of spinal cord from three groups on postoperative day 3 (scale bar= 25μm). (N) Quantification results of the related protein levels from (C), (G) and (K) in the indicated groups. The data are shown as the mean ± SEM. n = 5. *P < 0.05, **P < 0.01. ns indicates no significance. **Figure S8. **SS-31 facilitates functional recovery in SCI mice by inhibiting cPLA2 activity. (A, B, D) Photographs of spinal cord sections in the respective groups stained with MAP2 (green) and Syn (green)/NeuN (red). Relevant quantitative results for MAP2 optical density and motor neuron-contacting synapse amounts on day 28 after SCI. (C) Spinal cord longitudinal sections from the groups at 28 days after SCI were examined via HE dyeing and Masson dyeing (scale bar = 1000 μm). (E) Quantitative analyses of Masson-positive lesions within the spinal cord in the corresponding groups. (F) Photographs of mice footprints on day 28 following spinal cord injury. Forepaw print in blue; hindpaw print in red. (G, H) Stride length (cm) and toe dragging (%) analyses of mice at 28 dpi after SCI. (I-J) Inclined plane test and BMS for the corresponding groups at day 0, 1, 3, 7, 14, 21, and 28. The data are shown as the mean ± SEM. n = 5. *P < 0.05, **P < 0.01. ns indicates no significance. **Figure S9. **SS-31 protected lysosome function by inhibiting p38 signal pathway. (A-D) ELISA results of the activity of lysosomal enzymes CTSD and NAGLU in lysosomal (A-B) and cytosolic (C-D) fractions extracted from the spinal cord of SCI, SCI+SS-31, and SCI+SS-31+AA groups on postoperative day 3. The data are shown as the mean ± SEM. n = 5. *P < 0.05, **P < 0.01. ns indicates no significance.

## Data Availability

Data and materials are available under reasonable request.
