# Supplementary material for: Elamipretide alleviates pyroptosis in traumatically injured spinal cord by inhibiting cPLA2-induced lysosomal membrane permeabilization
Source: J Neuroinflammation. 2023 Jan 7;20:6. doi: 10.1186/s12974-023-02690-4 (PMC9825014; doi:10.1186/s12974-023-02690-4)
Supplement: Supplementary file 1 — Additional file 1: Figure S1. SS-31 does not affect the histological morphology and motor function in non-SCI mice. (A) Photographs of spinal cord sections in the Sham and Sham+SS-31 groups stained with antibody MAP2 (green) (scale bar = 25 μm). (B) MAP2 optical density within uninjured spinal cord on day 28. (C) Photographs of spinal cord sections in the respective groups stained with antibody Syn (green)/NeuN (red) (scale bar = 20 μm). (D) Relevant quantitative results for motor neuron-contacting synapse amounts on day 28 after sham surgery. (E) Photographs of mouse footprints on day 28 following sham surgery. Blue: forepaw print; Red: hind paw print. (F, G) Toe dragging (%) and stride length (cm) analyses of mice at 28 days after sham surgery. (H) Longitudinal spinal cord sections from the groups at 28 days after sham surgery were examined via HE dyeing and Masson dyeing (scale bar = 1000 μm). (I) Basso mouse scale (BMS) for the indicated groups at days 0, 1, 3, 7, 14, 21, and 28. The data are shown as the mean ± SEM. n = 5. *P < 0.05, **P < 0.01. ns indicates no significance. Figure S2. SS-31 attenuates pyroptosis in microglia after SCI. (A) Typical images of immunofluorescence staining for Caspase-1 and microglia colocalization and in the spinal cords of the Sham, SCI, and SCI+SS-31 groups (scale bar= 25μm) (B) Quantified Caspase-1 immunofluorescence data are presented on the right. (C) Typical images of immunofluorescence staining for NLRP3 and microglia colocalization and in the spinal cords of the Sham, SCI, and SCI+SS-31 groups (scale bar= 25μm) (D) Quantified NLRP3 immunofluorescence data are presented on the right. The white arrows indicate Caspase-1 or NLRP3-positive microglia. The data are shown as the mean ± SEM. n = 5. *P < 0.05, **P < 0.01. ns indicates no significance. Figure S3. CQ treatment does not affect pyroptosis in non-SCI mice. (A-D) Representative double immunofluorescence images for Caspase-1/NeuN and NLRP3/NeuN in spinal cord from Sham an [file 12974_2023_2690_MOESM1_ESM.docx]

**Additional Information**

**for**

**Elamipretide Alleviates Pyroptosis in Traumatically Injured Spinal Cords by Inhibiting cPLA2-Induced Lysosomal Membrane Permeabilization**

Haojie Zhang et. al

**Figure S1**


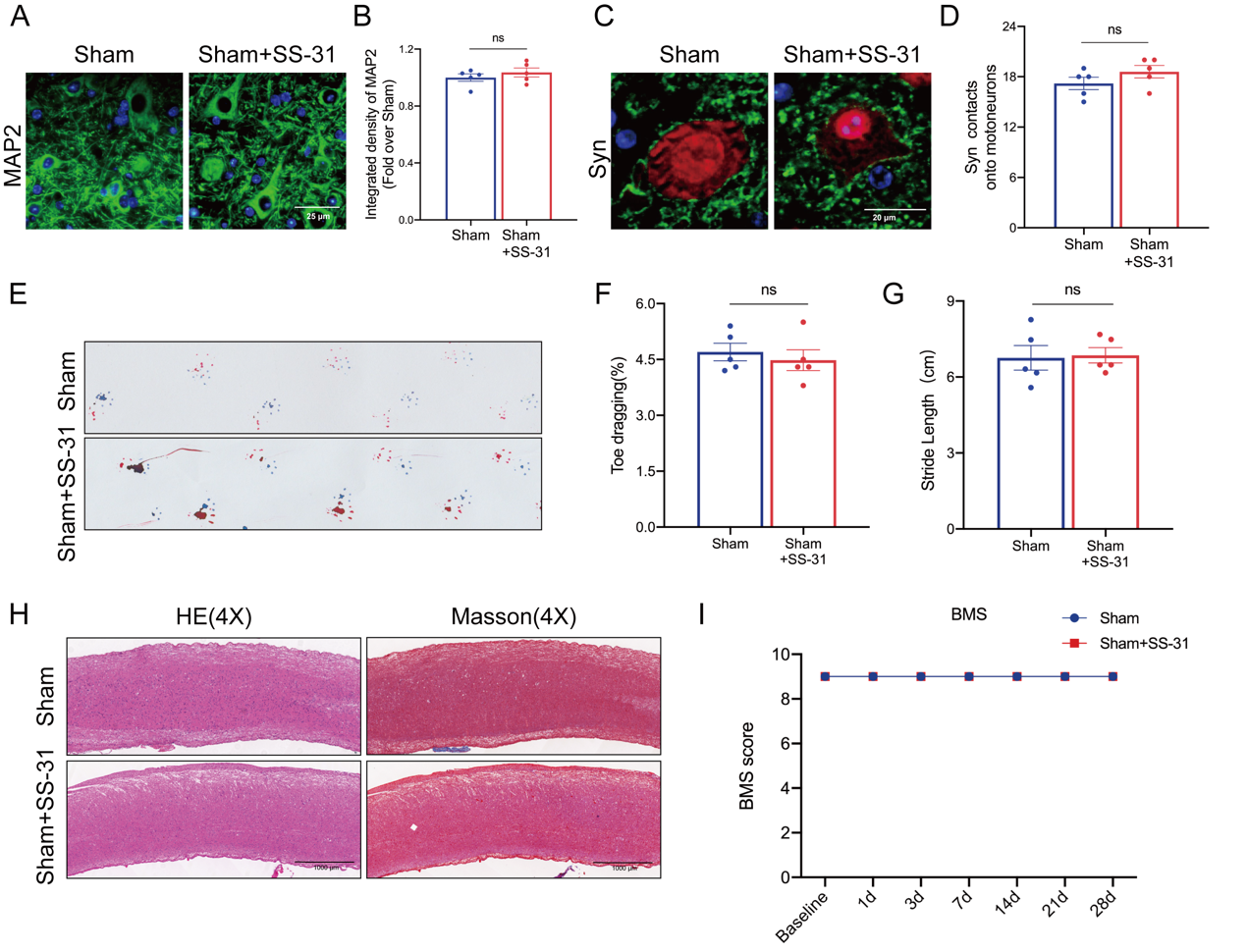


**Figure S1.**  SS-31doesn’t affect the histological morphology and motor function in non-SCI mice. (A) Photographs of spinal cord sections in the Sham and Sham+SS-31 groups stained with antibody MAP2 (green) (scale bar = 25 μm). (B) MAP2 optical density within uninjured spinal cord on day 28. (C) Photographs of spinal cord sections in the respective groups stained with antibody Syn (green)/NeuN (red) (scale bar = 20 μm). (D) Relevant quantitative results for motor neuron-contacting synapse amounts on day 28 after sham surgery. (E) Photographs of mouse footprints on day 28 following sham surgery. Blue: forepaw print; Red: hind paw print. (F, G) Toe dragging (%) and stride length (cm) analyses of mice at 28 days after sham surgery. (H) Longitudinal spinal cord sections from the groups at 28 days after sham surgery were examined via HE dyeing and Masson dyeing (scale bar = 1000 μm). (I) Basso mouse scale (BMS) for the indicated groups at days 0, 1, 3, 7, 14, 21, and 28. The data are shown as the mean ± SEM. n = 5. **P* < 0.05, ***P* < 0.01. ns indicates no significance.

**Figure S2**


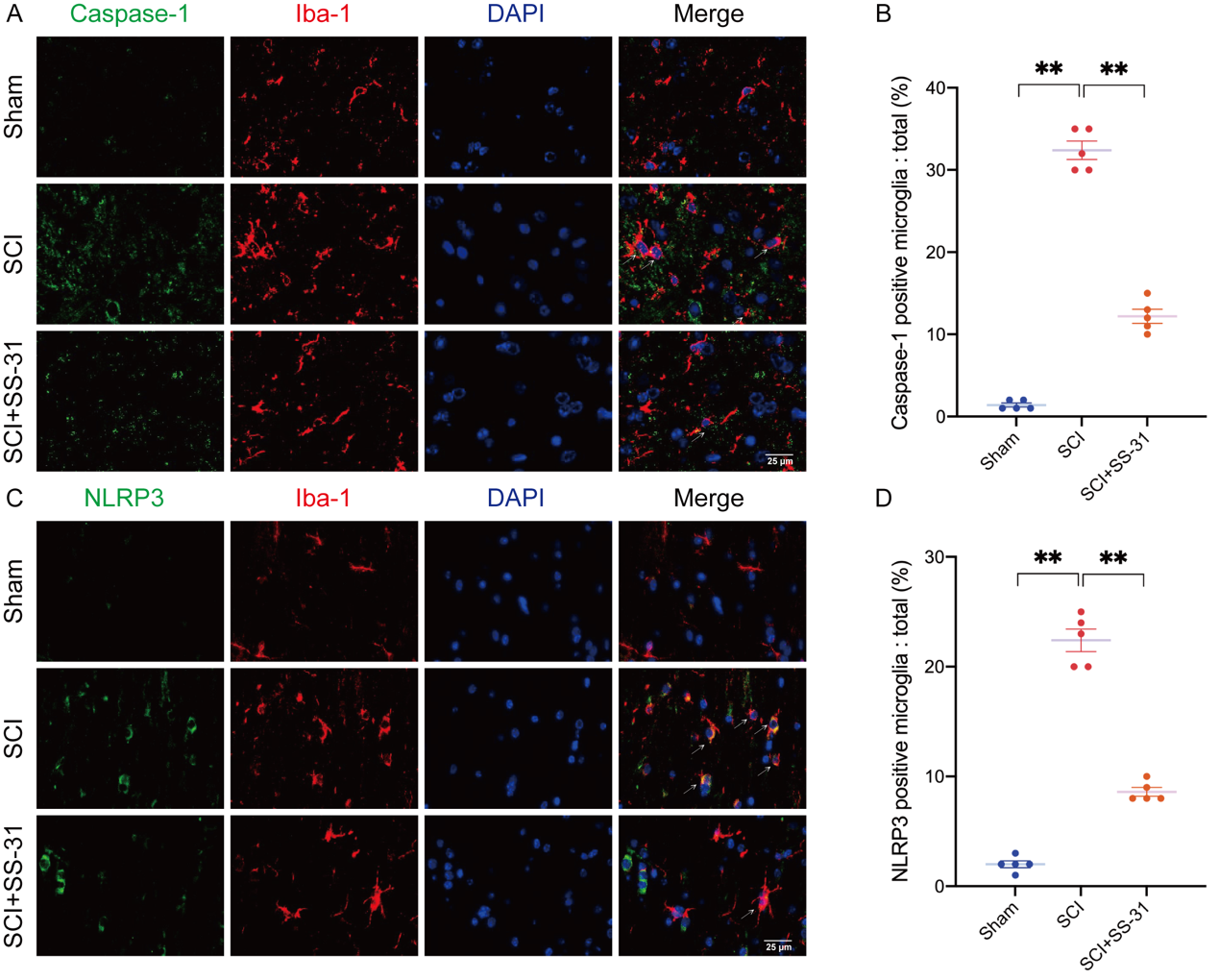


**Figure S2.** SS-31 attenuates pyroptosis in microglia after SCI. (A) Typical images of immunofluorescence staining for Caspase-1 and microglia colocalization and in the spinal cords of the Sham, SCI, and SCI+SS-31 groups (scale bar= 25μm) (B) Quantified Caspase-1 immunofluorescence data are presented on the right. (C) Typical images of immunofluorescence staining for NLRP3 and microglia colocalization and in the spinal cords of the Sham, SCI, and SCI+SS-31 groups (scale bar= 25μm) (D) Quantified NLRP3 immunofluorescence data are presented on the right. The white arrows indicate Caspase-1 or NLRP3 positive microglia. The data are shown as the mean ± SEM. n = 5. **P* < 0.05, ***P* < 0.01. ns indicates no significance.

**Figure S3**


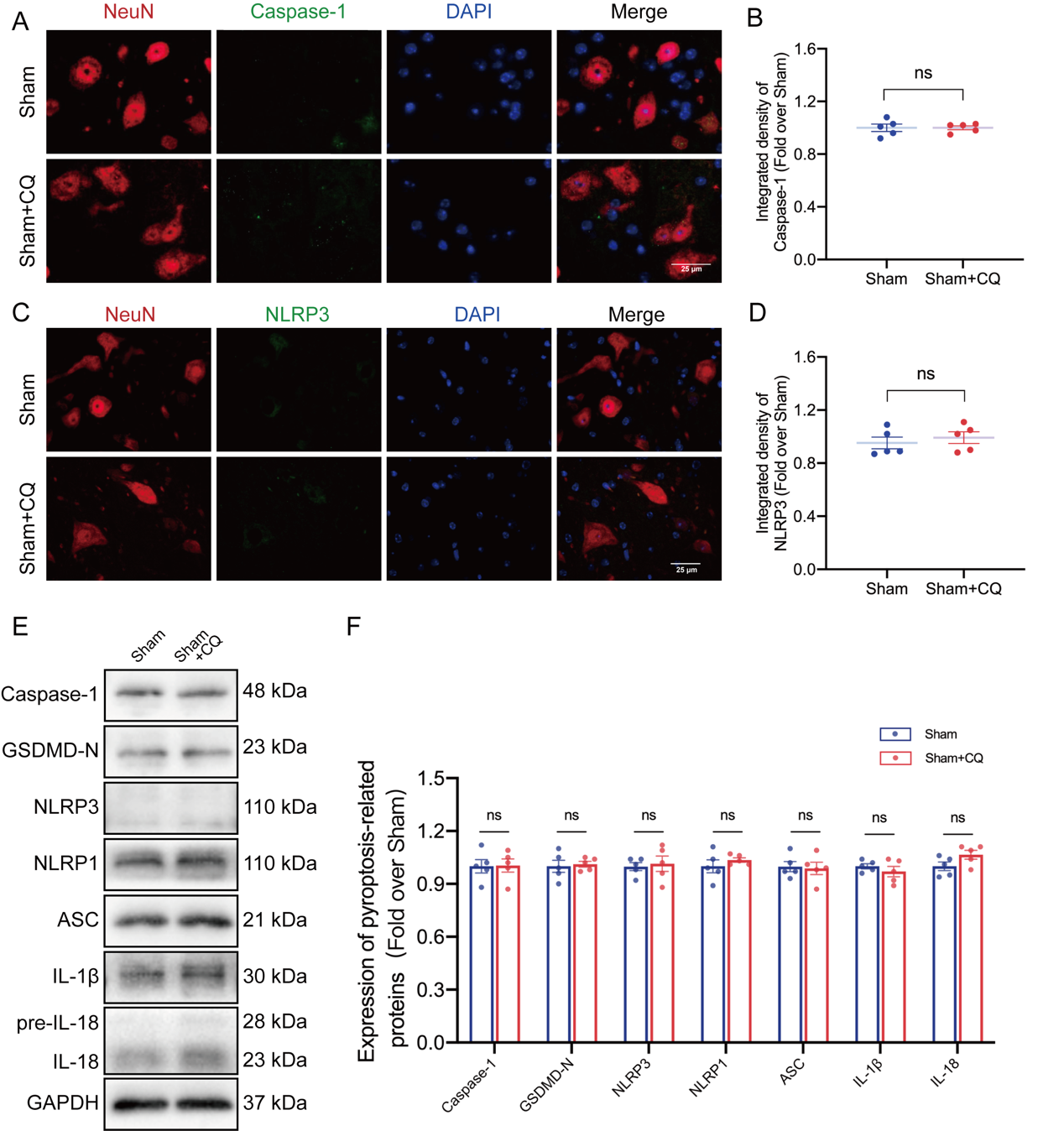


**Figure S3.** CQ treatment doesn’t not affect pyroptosis in non-SCI mice. (A-D) Representative double immunofluorescence images for Caspase-1/NeuN and NLRP3/NeuN in spinal cord from Sham and Sham+CQ groups at 3 dpi (scale bar= 25μm). The quantitative integrated densities of Caspase-1 and NLRP3 in each neuron are shown on the graph. (E) Typical images of WB results of Caspase-1, GSDMD-N, NLRP3, NLRP1, ASC, IL-1β, IL-18 in the spinal cord. GAPDH was utilized as a loading control. (F) Quantification results of the pyroptosis-related protein levels from (E) in both groups. The data are shown as the mean ± SEM. ns indicates no significance.

**Figure S4**


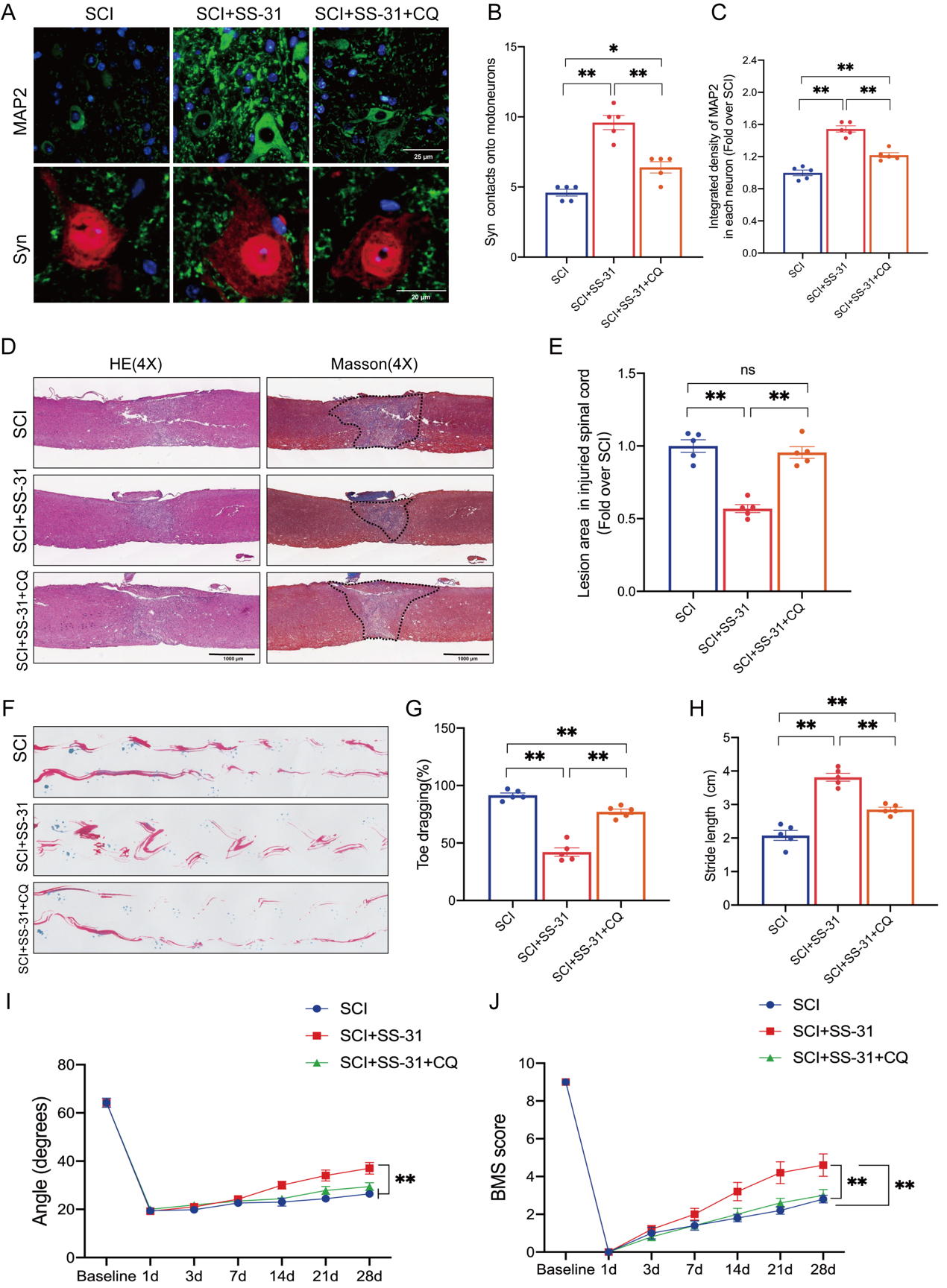


**Figure S4.** SS-31’s functional recovery effect is inhibited by CQ. (A) Photographs of spinal cord sections in the SCI, SCI+SS-31, SCI+SS-31+CQ groups stained with antibody MAP2 (green) (scale bar = 25 μm). Photographs of spinal cord sections in the respective groups stained with Syn (green)/NeuN (red) (scale bar = 20 μm). (B) Relevant quantitative results for motor neuron-contacting synapse amounts on day 28 after SCI. (C) MAP2 optical density within a spinal cord subjected to injury on day 28. (D) Longitudinal spinal cord sections at 28 dpi after SCI stained with HE dyeing and Masson dyeing (scale bar = 1000 μm). (E) The lesion area in injured spinal cord measured from Masson staining. (F) Photographs of mouse footprints on day 28 following spinal cord injury. Blue: forepaw print; Red: hind paw print. (G, H) Toe dragging (%) and stride length (cm) analyses of mice at 28 dpi after SCI. (I, J) Inclined plane test and Basso mouse scale (BMS) for the indicated groups at days 0, 1, 3, 7, 14, 21, and 28. The data are shown as the mean ± SEM. n = 5. **P* < 0.05, ***P* < 0.01. ns indicates no significance.

**Figure S5**


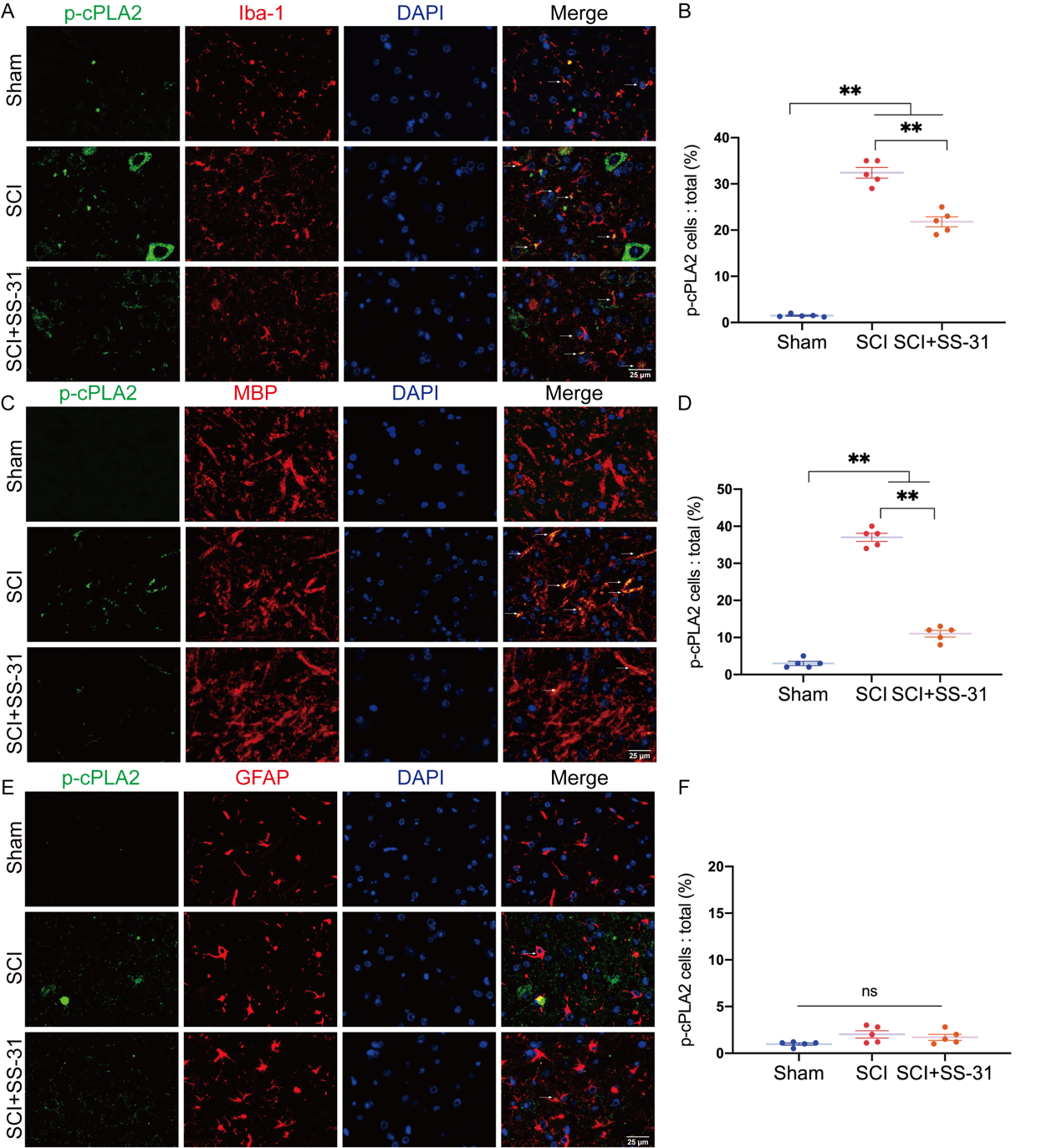


**Figure S5.** SS-31 affectes the p-cPLA2 expression in glial cells. (A-B) Typical images of immunofluorescence staining for p-cPLA2 and microglia colocalization in the spinal cords of the Sham, SCI, and SCI+SS-31 groups. The white arrow indicates the p-cPLA2 positive microglia. The ratio of p-cPLA2 positive cells to the total is shown on the right. (C-D) Typical images of immunofluorescence staining for p-cPLA2 and oligodendrocyte colocalization in the spinal cords of three groups. The white arrow indicates the p-cPLA2 positive oligodendrocyte. The ratio of p-cPLA2 positive cells to the total is shown on the right. (E-F) Typical images of immunofluorescence staining for p-cPLA2 and astrocyte colocalization in the spinal cords of three groups. The white arrow indicates the p-cPLA2 positive astrocyte. The ratio of p-cPLA2 positive cells to the total is shown on the right. The data are shown as the mean ± SEM. n = 5. **P* < 0.05, ***P* < 0.01. ns indicates no significance.

**Figure S6**


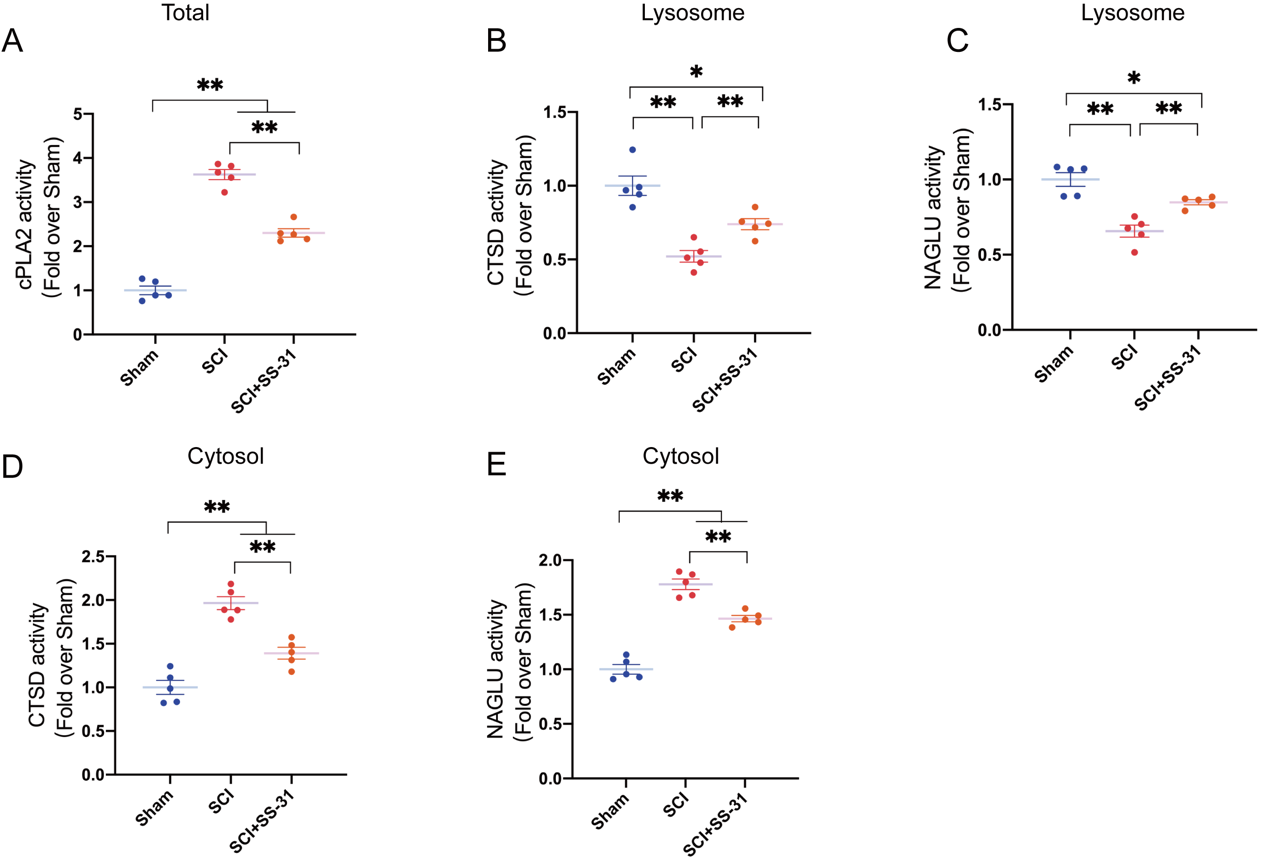


**Figure S6.** SS-31 inhibits the activity of cPLA2 and protect the lysosomal enzymes activity in lysosomes. (A) ELISA results showing the activity of cPLA2 in the spinal cord from Sham, SCI, and SCI+SS-31 groups on postoperative day 3. (B-D) ELISA results of the activity of lysosomal enzymes CTSD and NAGLU in lysosomal (B, C) and cytosolic (D, E) fractions extracted from the spinal cord of Sham, SCI, and SCI+SS-31 groups on postoperative day 3. The data are shown as the mean ± SEM. n = 5. **P* < 0.05, ***P* < 0.01. ns indicates no significance.

**Figure S7**


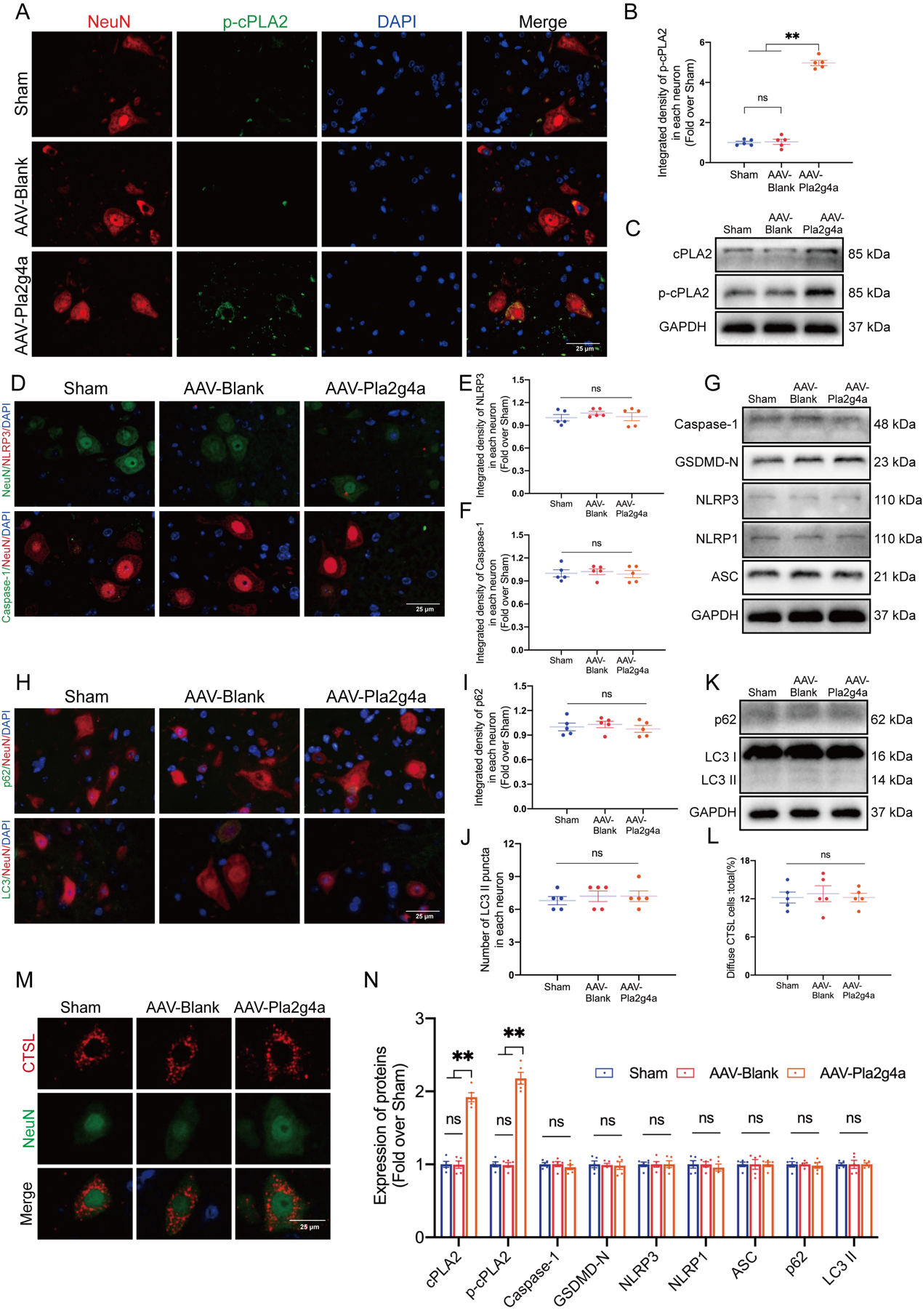


**Figure S7.** Pla2g4a overexpression doesn’t affect pyroptosis, autophagy or LMP in non-SCI mice. (A-B) Representative immunofluorescence images of p-cPLA2 and NeuN in the anterior horn areas of spinal cord from Sham, Sham+AAV-Blank, Sham+AAV-Pla2g4a groups after 3 dpi. (scale bar= 25μm). The quantification data was showed on the right. (C) cPLA2 and p-cPLA2 protein levels in the spinal cord from three groups on postoperative day 3. (D-F) Representative double immunofluorescence images for NLRP3/NeuN and Caspase-1/NeuN in spinal cord from three groups at 3 dpi (scale bar= 25μm). The quantitative integrated densities of NLRP3 and Caspase-1 in each neuron are shown on the graph. (G) Typical images of WB analyses of Caspase-1, GSDMD-N, NLRP3, NLRP1, ASC in the spinal cord. GAPDH was utilized as a loading control. (H-J) Representative double immunofluorescence images for p62/NeuN and LC3/NeuN in spinal cord lesions from three groups at 3 dpi (scale bar= 25μm). The number of LC3 II puncta and the quantitative integrated densitiy of p62 in each neuron are shown on the graph. (K) Typical images of WB analyses of p62 and LC3 in the spinal cord. GAPDH was utilized as a loading control. (L) Comparison of the ratio of diffuse CTSL cells in the anterior horn of spinal cord among three groups. (M) Immunofluorescence staining of NeuN and CTSL in the anterior horn of spinal cord from three groups on postoperative day 3 (scale bar= 25μm). (N) Quantification results of the related protein levels from (C), (G) and (K) in the indicated groups. The data are shown as the mean ± SEM. n = 5. **P* < 0.05, ***P* < 0.01. ns indicates no significance.

**Figure S8**


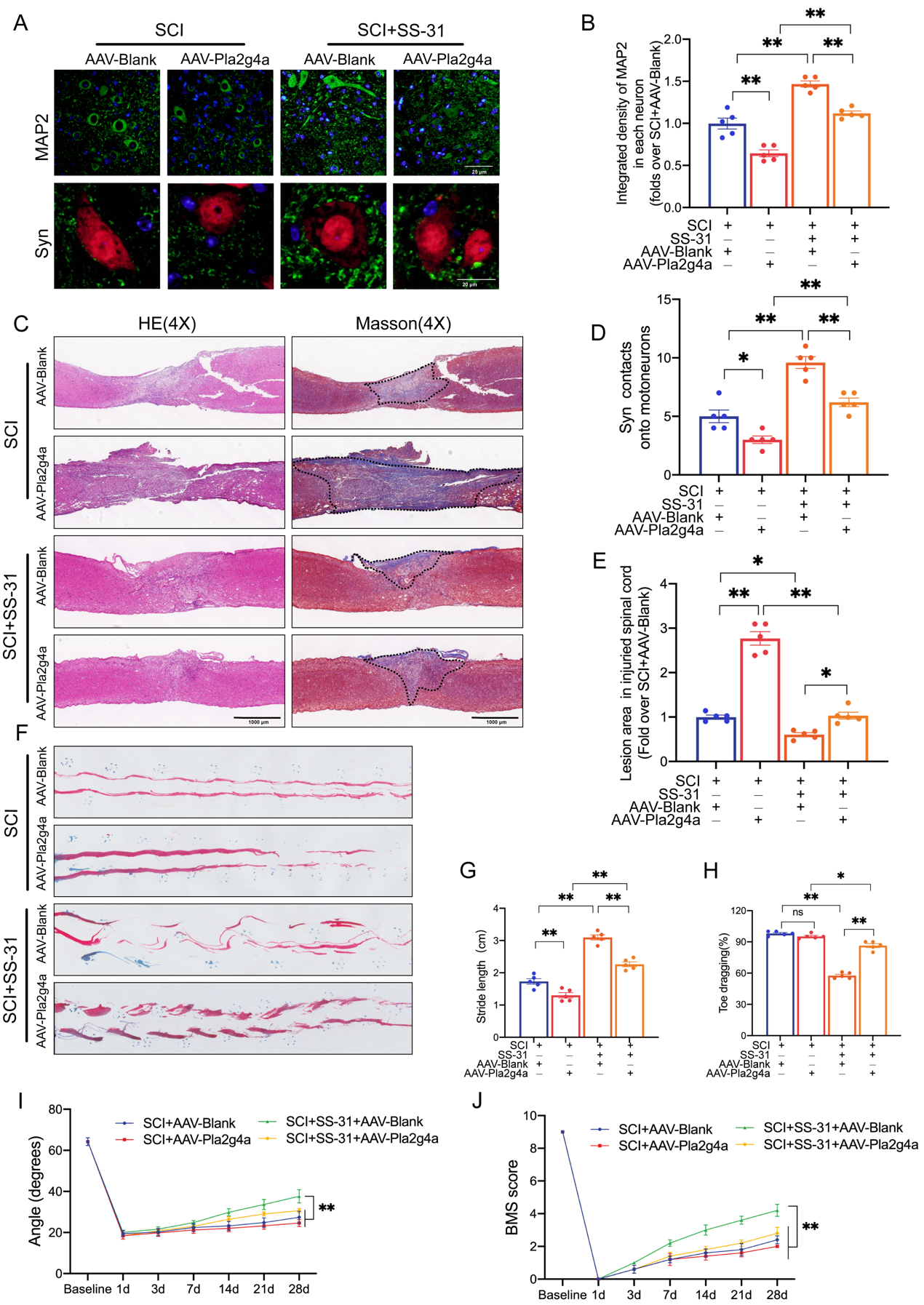


**Figure S8.** SS-31 facilitats functional recovery in SCI mice by inhibiting cPLA2 activity. (A, B, D) Photographs of spinal cord sections in the respective groups stained with MAP2 (green) and Syn (green)/NeuN (red). Relevant quantitative results for MAP2 optical density and motor neuron-contacting synapse amounts on day 28 after SCI. (C) Spinal cord longitudinal sections from the groups at 28 days after SCI were examined via HE dyeing and Masson dyeing (scale bar = 1000 μm). (E) Quantitative analyses of Masson-positive lesions within the spinal cord in the corresponding groups. (F) Photographs of mice footprints on day 28 following spinal cord injury. Forepaw print in blue; hindpaw print in red. (G, H) Stride length (cm) and toe dragging (%) analyses of mice at 28 dpi after SCI. (I-J) Inclined plane test and BMS for the corresponding groups at day 0, 1, 3, 7, 14, 21, and 28. The data are shown as the mean ± SEM. n = 5. **P* < 0.05, ***P* < 0.01. ns indicates no significance.

**Figure S9**


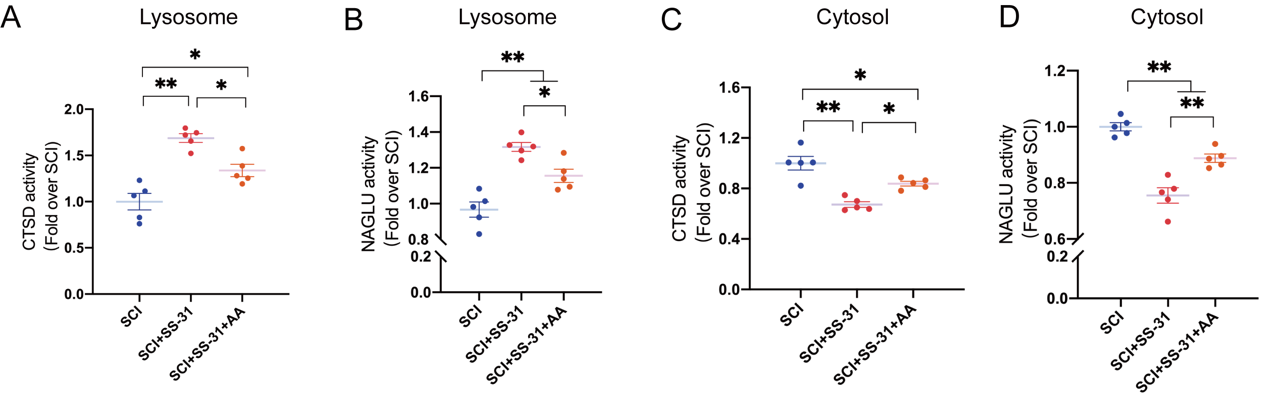


**Figure S9.** SS-31 protected lysosome function by inhibiting p38 signal pathway. (A-D) ELISA results of the activity of lysosomal enzymes CTSD and NAGLU in lysosomal (A-B) and cytosolic (C-D) fractions extracted from the spinal cord of SCI, SCI+SS-31, and SCI+SS-31+AA groups on postoperative day 3. The data are shown as the mean ± SEM. n = 5. **P* < 0.05, ***P* < 0.01. ns indicates no significance.
